# The Cost-Effectiveness of Three Prevention Strategies in Alzheimer's Disease: Results from the Multidomain Alzheimer Preventive Trial (MAPT)

**DOI:** 10.14283/jpad.2021.47

**Published:** 2021-08-02

**Authors:** Nadège Costa, M. Mounié, A. Pagès, H. Derumeaux, T. Rapp, S. Guyonnet, N. Coley, C. Cantet, I. Carrié, S. Andrieu, L. Molinier, MAPT/DSA Group

**Affiliations:** 1 Health Economic Unit of the University Hospital of Toulouse, Hôtel Dieu Saint-Jacques, 2, rue viguerie, 31059, Toulouse Cedex 9, France; 2 INSERM-UMR 1027, 31000, Toulouse, France; 3 University of Toulouse III, 31330, Toulouse, France; 4 Department of Pharmacy, Toulouse University Hospital, 31000, Toulouse, France; 5 Gérontopôle, Department of Geriatrics, University Hospital of Toulouse, 31059, Toulouse, France; 6 Université de Paris (LIRAES EA4470), Paris, 75006, Paris, France; 7 Department of Epidemiology and Public Health, university Hospital of Toulouse, 31059, Toulouse, France

**Keywords:** Cost-effectiveness, economics, Alzheimer disease, prevention

## Abstract

**Background:**

To date, no curative treatment is available for Alzheimer's disease (AD). Therefore, efforts should focus on prevention strategies to improve the efficiency of healthcare systems.

**Objective:**

Our aim was to assess the cost-effectiveness of three preventive strategies for AD compared to a placebo.

**Design:**

The Multidomain Alzheimer Preventive Trial (MAPT) study was a multicenter, randomized, placebo-controlled superiority trial with four parallel groups, including three intervention groups (one group with Multidomain Intervention (MI) plus a placebo, one group with Polyunsaturated Fatty Acids (PFA), one group with a combination of PFA and MI) and one placebo group.

**Setting:**

Participants were recruited and included in 13 memory centers in France and Monaco.

**Participants:**

Community-dwelling subject aged 70 years and older were followed during 3 years.

**Interventions:**

We used data from the MAPT study which aims to test the efficacy of a MI along PFA, the MI plus a placebo, PFA alone, or a placebo alone.

**Measurement:**

Direct medical and non-medical costs were calculated from a payer's perspective during the 3 years of follow-up. The base case incremental Cost-Effectiveness Ratio (ICER) represents the cost per improved cognitive Z-score point. Sensitivity analyses were performed using different interpretation of the effectiveness criteria.

**Results:**

Analyses were conducted on 1,525 participants. The ICER at year 3 that compares the MI + PFA and the MI alone to the placebo amounted to €21,443 and €21,543 respectively, per improved Z score point. PFA alone amounted to €111,720 per improved Z score point.

**Conclusion:**

Our study shows that ICERS of PFA combined with MI and MI alone amounted to €21,443 and €21,543 respectively per improved Z score point compared to the placebo and are below the WTP of €50,000 while the ICER of PFA alone amounted to €111,720 per improved Z score point. This information may help decision makers and serve as a basis for the implementation of a lifetime decision analytic model.

## Introduction

**A**ccording to the 2019 World Alzheimer report, 50 million people worldwide and 1.2 millions in France suffer from Alzheimer's disease (AD) (1). Associated costs of care are consistent and vary from €24,140 for mild and moderate stages to €44,171 for the severe stage at 18 months (2).

According to the latest meta-analyses, specific drugs in the treatment of AD have a low and time-limited efficacy on symptoms, quality of life, institutionalization, mortality and the burden of caregivers (3, 4). In 2016, the French High Authority for Health (HAS) considered that the benefit of these medicines was insufficient to justify reimbursement by the French National Health Insurance (FNHI) (5, 6).

As no curative treatment is available, efforts should focus on prevention strategies. Current evidence suggests that nutrition, physical exercise, cognitive activity and social stimulation may improve cognitive health (7). Results from the Multidomain Alzheimer Preventive Trial (MAPT), which test the effect of Multidomain Intervention (MI) and supplementation using omega 3 polyunsaturated fatty acids (PFA) alone or in combination on cognitive decline alongside a large randomized controlled trial show no significant differences in 3-year cognitive decline between any of the three intervention groups and the placebo group (8). Nevertheless, this trial shows a trend in z-score differences in favor of MI + PFA and MI alone groups.

Published cost-effectiveness analyses of primary prevention strategies for AD show cost-saving results. Nevertheless, these studies are only based on simulated models and hypothetical interventions indicating potential cost-effectiveness results (9). Interventions tested were physical activity, management of cardiovascular risk factors, vitamin supplementation, and multidomain cardiovascular disease prevention programs (10, 11, 12, 13). Currently in France, these interventions are not reimbursed specifically for the prevention of Alzheimer's disease but they can be offered to the patient for the maintenance of their overall health. More randomized control trials (RCT) are required to reinforce the results cost-effectiveness study of prevention programs for AD.

In the framework of the large MAPT study, we aim to assess the cost-effectiveness of PFA supplementation alone, MI (nutritional counseling, physical exercise, and cognitive stimulation) alone or a combination of both interventions compared to a placebo.

## Methods

### Design, setting and participants

The MAPT study was a multicenter, randomized, placebo-controlled superiority trial with four parallel groups, including three intervention groups (one group with MI plus a placebo, one group with PFA, one group with a combination of PFA and MI) and one placebo group. Community-dwelling subjects, followed during 3 years, aged 70 years and older were recruited at 13 memory centers in France and Monaco. In France, memory centers are outpatient structures that performed diagnostic workup and follow-up of elderly subjects. Participants met at least one of three criteria: spontaneous memory complaint, limitation in one instrumental Activity of Daily Living (ADL), or slow gait were eligible to be included in the study. Participants with a Mini Mental State Examination (MMSE) score below 24, those who were diagnosed with dementia, those with any difficulty in basic ADL and those taking PFA supplements at baseline were excluded. Full methods have been previously described elsewhere (8, 14). The trial protocol was approved by the French Ethics Committee in Toulouse (CPP SOOM II) and was authorized by the French Health Authority (8).

### Interventions

Participants were randomly assigned to one of the following four groups:

• Multidomain Intervention: consisted of 2 h group sessions focusing on three domains (cognitive stimulation, physical activity, and nutrition) and a preventive consultation (at baseline, 12 months, and 24 months). MI was done twice a week during the first month, once a week during the second month, and one per month for the remainder of the three years study,
• Omega 3 Polyunsaturated Fatty Acids: two capsules per day with 400 mg docosahexaenoic acid (DHA) and no more than 112.5 mg eicosapentaenoic acid (EPA),
• Combined intervention: Multidomain intervention and Omega-3 PFA,
• Placebo: two capsules per day containing flavoured paraffin oil.

More details are given elsewhere (8).

### Outcomes

All costs were recorded throughout the MAPT trial at 6, 12, 18, 24, 30 and 36 months using a Case Report Form and were analysed from the FNHI perspective. All monetary values are in 2018 Euros. Costs taken into account were direct medical (hospitalizations, consultations, medical and paramedical procedures and drugs) and non-medical (transportation) costs.

Valuation was based on several sources of unit costs (Appendix 1. Table A1). Hospital stays were valued using the French Disease Related Groups (DRG) including extra charges if applicable (e.g. the cost of days of intensive care) (15). We used mean DRG rates calculated from the national hospitalization database for patients aged 70 or over, according to the medical unit to which the participant was admitted. Rehabilitation and psychiatric hospitalizations were valued using per diem costs. Consultations were valued using the General French Nomenclature for Medical Procedures according to the specialization (16). Medical procedures were valued using the Medical Classification of Clinical Procedures (CCAM) (Version 54.10) and the Nomenclature of Clinical Biological Procedures (NABM) according to the type of medical procedure (imaging, biology, other) (17, 18). Each consultation and medical procedure was valued using the appropriate FNHI reimbursement rate.

No details, except the frequency, were available in the database on transportation and paramedical procedures, therefore valuation was based on means estimate from a sample of the FNHI database, the General Sample of Beneficiaries database (EGB) (19, 20). The gamma distribution shape and scale parameters were derived from the mean and variance observed in the 2018 EGB database for each cost component for the population aged 70 years or older.

For drugs reimbursed by the FNHI, we assumed that the daily dosage was equal to the Daily Defined Dose (DDD) (21). If there was no recommended DDD, we calculated an average daily dose according to the Summary of Product Characteristics (SmPC) (22). We then multiplied the reimbursement price per unit by the daily dosage and the treatment duration (23). For hospital drugs, only the costs of very expensive drugs were taken into account because the others were included in the DRG rate (24).

MI was valued by the mean wage rate for a psychologist, dietician and physical activity facilitator (40€) multiplied by the intervention period (2.30 hours) and the number of prescribed sessions (46) during three years. PFA was valued using the retail price per capsule (€0.50 cents) multiplied by the number of prescribed capsules taken per participant per year (365.5/year), multiplied by 3 years.

The primary efficacy outcome used to determine the ICER consisted of the change from baseline to 36 months in a composite Z score (8). It combines four cognitive tests (free and total recall of the Free and Cued Selective Reminding Test, ten MMSE orientation items, the Digit Symbol Substitution Test score from the Revised Wechsler Adult Intelligence Scale, and the Category Naming Test [2 min category fluency in animals]) (8). Z-scores represent the number of standard deviations above or below the mean. Coley et al estimated that a 0.3-point decrease in Z score is the minimum clinically significant difference, which predicts dementia (25). We used this cut-off, in addition to the Z-score, to define whether a participant presented an aggravation in memory function in order to make the ICER more comprehensive for clinicians and decision makers. Other variables (age, gender, comorbidities, Fried frailty phenotype, educational level and Z score) were collected at baseline.

### Statistical analyses

Description and comparison of baseline characteristics were made using mean and standard deviation and occurrences and percentages for continuous and qualitative variables on one hand and using Kruskal Wallis or Chi-squared on the other hand.

Cost components for participants who had a complete follow-up were summarized for each group. Three-year cumulative costs were expressed in terms of mean costs per participant and their bias-corrected and accelerated (BCa) bootstrap 95% confidence intervals (CI). Cost differences between groups where tested using a global non-parametric Kruskal-Wallis test.

Missing data on total cumulative cost at 3 years were accounted for by multiple imputation and predictive mean matching methods (26). Age group tercile, gender, intervention groups, initial frailty score tercile and pooled occurrences of medical history tercile were used in the imputation. We assumed that missing cost data are “Missing At Random” and we used Hausman test to verify whether our results were subject to attrition bias issues (27). Efficacy data used in our analysis were smoothed by a mixed model as described elsewhere (8). The fixed effects used in this model were intervention group, time, and interactions between intervention groups and each time. The random effects used were center-specific and participant-specific variables. In order to include adjusted outcomes in both the numerator and denominator of the ICER, we used a Generalized Linear Model (GLM) with a gamma distribution and a log link that allowed the use of fitted cost data (28). The same variables used for imputation were also used for adjustment. Fitted and imputed costs as well as fitted Z scores were then described using mean and BCA bootstrap CI.

We used non-parametric bootstrap outputs to graphically determined the 95% confidence ellipses and illustrate the uncertainty around the ICER (29, 30). ICERs with a positive value were compared to a Willingness-To-Pay (WTP) threshold set up at 50,000€ per Quality Adjusted Life Years (31, 32, 33). Additionally, the cost-effectiveness acceptability curve (CEAC) showed the probability that an intervention was cost-effective compared with the alternative according to a range of WTP thresholds (34). Moreover, sensitivity analysis was conducted using the data for patient with a complete follow-up.

## Results

### Patient's characteristics

**Figure 1:**
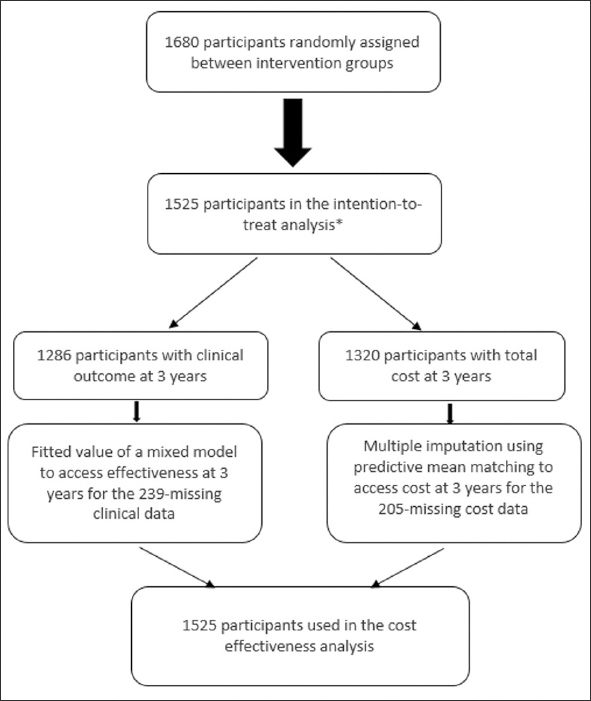
Flow chart for patient's selection *The intention-to-treat analysis included assigned participants with a composite score at baseline who had at least one post-baseline visit.

Between 30 May 2008 and 24 February 2011, 1,680 participants were enrolled and randomly assigned to four arms. Participants were excluded from the modified intention-to-treat efficacy analysis because no cognitive assessment was available after baseline for 154 participants, and one participant in the PFA group withdrew their consent. One thousand two hundred and eighty-six participants completed the final visit and economic data were available for 1,320 participants (Figure 1). Missing economic data accounted for 12% to 15% in each group. A full description of the population was provided in prior work (14). The baseline characteristics of our sample are summarized in Table 1. No substantial difference was noted in any demographic or clinical characteristics between the arms.

**Table 1:** Participants' characteristics at baseline

N: 1525	PFA§ + MI ∥	PFA §	MI ∥	Placebo	p*
Number of participants, n (%)	374 (24.52)	381 (24.98)	390 (25.57)	380 (24.92)	-
Age, mean (sd)	75.4 (4.4)	75.6 (4.7)	75 (4.1)	75.1 (4.3)	0.12
Gender (male), n (%)	145 (38.77)	136 (35.7)	138 (35.38)	128 (33.68)	0.53
Initial Z score, mean (sd)	−0.043 (0.676)	0.028 (0.626)	−0.002 (0.713)	0.018 (0.662)	0.70
Frail status, n (col %)					
Robust	214 (57.22)	201 (52.76)	225 (57.69)	220 (57.89)	0.21
Pre-frail	148 (39.57)	165 (43.31)	153 (39.23)	156 (41.05)	
Frail	12 (3.21)	15 (3.94)	12 (3.08)	4 (1.05)	
Educational level, n (col %)					
No diploma or primary school certificate	75 (20.05)	96 (25.2)	81 (20.77)	82 (21.58)	0.14
Secondary education	145 (38.77)	120 (31.5)	129 (33.08)	117 (30.79)	
High school diploma	52 (13.9)	43 (11.29)	56 (14.36)	67 (17.63)	
University level	100 (26.74)	108 (28.35)	120 (30.77)	110 (28.95)	
Missing	2 (0.53)	14 (3.67)	4 (1.03)	4 (1.05)	
Medical history, n (col %)					
Skin diseases	29 (7.75)	32 (8.4)	29 (7.44)	32 (8.42)	0.94
GI diseases †	142 (37.97)	145 (38.06)	145 (37.18)	156 (41.05)	0.70
Genitourinary diseases	105 (28.07)	110 (28.87)	114 (29.23)	93 (24.47)	0.44
Respiratory diseases	8 (2.14)	13 (3.41)	20 (5.13)	16 (4.21)	0.16
Eye disease	54 (14.44)	61 (16.01)	59 (15.13)	62 (16.32)	0.89
ENT disease ‡	102 (27.27)	85 (22.31)	89 (22.82)	99 (26.05)	0.31
Blood and immune diseases	24 (6.42)	24 (6.3)	24 (6.15)	25 (6.58)	0.10
Nervous system diseases	119 (31.82)	121 (31.76)	120 (30.77)	122 (32.11)	0.98
Musculoskeletal diseases	262 (70.05)	283 (74.28)	287 (73.59)	276 (72.63)	0.58
Endocrine, nutritional and metabolic diseases	226 (60.43)	242 (63.52)	238 (61.03)	231 (60.79)	0.81
Infectious diseases	79 (21.12)	61 (16.01)	69 (17.69)	62 (16.32)	0.24
Mental and behavioral disorders	100 (26.74)	104 (27.3)	102 (26.15)	103 (27.11)	0.98
Cancers	115 (30.75)	115 (30.18)	115 (29.49)	98 (25.79)	0.43
Cardiovascular diseases	281 (75.13)	280 (73.49)	298 (76.41)	284 (74.74)	0.83
Benign tumors	51 (13.64)	44 (11.55)	64 (16.41)	52 (13.68)	0.28


*p-value of khi2 or Kruskal Wallis test according to variables; † Gastrointestinal; ‡Ear Nose and Throat; §Polyunsaturated Fatty Acids; ∥ Multidomain intervention

### Three years costs description

The observed costs for the three-year follow-up period for 1,320 participants with complete economic data are presented in table 2. Total costs without intervention amounted to €7,702; €7,951; €7,845 and €7,106 for PFA + MI, PFA, MI and the placebo group, respectively (p=0.77). When the intervention cost was included in the total costs, they were significantly different and amounted to €9,171; €8,500; €8,765 and €7,106, respectively (p=0.001). The main cost driver in each group was inpatient stays which accounted for approximately 50% of the total cost in all groups. The second cost driver was medication, which accounted for 24% to 30% of the total cost depending on the group.

**Table 2:** Total costs over the three-year follow-up period

	PFA§ + MI∥		PFA§		MI∥		Control		
	Frequency	Cost	Frequency	Cost	Frequency	Cost	Frequency	Cost	p†
	Mean	Mean	Mean	Mean	Mean	Mean	Mean	Mean	
	[95% CI*]	[95%, CI*]	[95%, CI*]	[95%, CI*]	[95%, CI*]	[95%, CI*]	[95%, CI*]	[95%, CI*]	
Inpatient stays	1.03	4358	1.08	4385	1.01	4328	0.95	3829	0.70
	[0.9; 1.19]	[3518; 5585]	[0.91; 1.44]	[3447; 6072]	[0.85; 1.2]	[3384; 5384]	[0.83; 1.11]	[3237; 4737]	
MSO‡ <1 day	0.29	297	0.41	378	0.35	358	0.33	336	0.83
	[0.22; 0.36]	[226; 370]	[0.3; 0.78]	[293; 510]	[0.28; 0.49]	[279; 467]	[0.27; 0.42]	[268; 428]	
MSO‡ ≥1 day	0.67	2911	0.61	2655	0.58	2473	0.56	2569	0.18
	[0.57; 0.8]	[2488; 3489]	[0.51; 0.74]	[2200; 3186]	[0.45; 0.71]	[1990; 3032]	[0.47; 0.66]	[2185; 3009]	
Rehabilitation	0.04	692	0.04	850	0.05	866	0.06	774	0.76
	[0.02; 0.08]	[348; 1316]	[0.02; 0.08]	[377; 1868]	[0.03; 0.08]	[478; 1484]	[0.03; 0.1]	[405; 1382]	
Psychiatric unit care	0.02	458	0.01	502	0.02	631	0.01	151	0.70
	[0.01; 0.05]	[84; 1543]	[0; 0.04]	[0; 2169]	[0.01; 0.05]	[201; 1608]	[0; 0.02]	[0; 519]	
Emergency Room	0.07	13	0.06	12	0.04	7	0.05	9	0.57
	[0.04; 0.11]	[7; 21]	[0.03; 0.1]	[6; 19]	[0.02; 0.06]	[3; 12]	[0.02; 0.08]	[5; 14]	
Consultation	25.54	454	29.03	516	26.58	470	27.95	495	0.012
	[24.25; 27.07]	[428; 478]	[27.31; 30.65]	[488; 549]	[24.96; 28.3]	[439; 500]	[26.24; 29.88]	[463; 537]	
General practitioner	15.04	248	16.49	272	14.85	245	15.05	248	0.026
	[14.15; 16.07]	[232; 265]	[15.58; 17.5]	[257; 290]	[14.02; 15.92]	[230; 261]	[14.1; 16.07]	[233; 264]	
Specialist	10.51	206	12.54	244	11.73	225	12.9	247	0.24
	[9.64; 11.36]	[191; 224]	[11.5; 14.08]	[223; 269]	[10.67; 13.03]	[205; 251]	[11.6; 14.45]	[226; 282]	
Cardiologist	1.8	58	1.98	64	1.6	52	1.59	52	0.018
	[1.59; 2.06]	[51; 67]	[1.71; 2.26]	[57; 74]	[1.39; 1.89]	[45; 61]	[1.35; 1.87]	[45; 61]	
Psychiatrist & Neurologist	0.42	11	0.53	14	0.67	18	0.92	24	0.81
	[0.3; 0.65]	[7; 16]	[0.31; 1.07]	[8; 30]	[0.39; 1.07]	[11; 29]	[0.51; 2.19]	[14; 53]	
Rheumatologist	1.78	29	2	33	2.01	33	1.98	33	0.52
	[1.49; 2.16]	[24; 36]	[1.69; 2.45]	[28; 40]	[1.67; 2.5]	[27; 42]	[1.68; 2.38]	[28; 39]	
Ophthalmologist	1.88	31	1.86	31	2.06	34	2.17	36	0.74
	[1.66; 2.16]	[27; 36]	[1.61; 2.14]	[27; 35]	[1.72; 2.51]	[29; 43]	[1.85; 2.59]	[31; 43]	
Other specialists	4.62	76	6.17	102	5.39	89	6.24	103	0.023
	[4.12; 5.24]	[67; 86]	[5.34; 7.29]	[89; 119]	[4.65; 6.5]	[77; 107]	[5.4; 7.33]	[91; 122]	
Paramedical procedures	22.12	425	34.72	598	21.14	357	21.81	393	0.09
	[17.46; 29.06]	[328; 583]	[21.72; 74.7]	[402; 1304]	[17.24; 26.4]	[291; 442]	[18.8; 25.52]	[330; 492]	
Nurse	3.85	127	9.19	243	2.95	57	2.59	66	0.73
	[2.39; 6.23]	[66; 265]	[2.61; 24.65]	[66; 1165]	[1.99; 4.38]	[34; 108]	[1.86; 3.77]	[40; 146]	
Physiotherapist	14.41	288	16.34	330	15.25	293	16.64	321	0.11
	[11.69; 17.98]	[231; 367]	[13.38; 19.98]	[268; 411]	[11.93; 19.14]	[234; 362]	[13.9; 19.8]	[270; 395]	
Other	3.85	10	9.19	25	2.95	8	2.59	7	0.77
	[2.5; 6.77]	[7; 19]	[2.79; 33.43]	[8; 73]	[2.02; 4.45]	[5; 12]	[1.84; 3.73]	[5; 10]	
Medical procedures	24.41	205	24.77	245	22.84	195	22.06	228	0.016
	[22.18; 27.16]	[186; 232]	[22.57; 27.28]	[222; 269]	[20.96; 24.97]	[176; 222]	[20.13; 24.51]	[205; 255]	
Laboratory tests	21.16	93	21.06	116	19.84	87	18.32	103	0.009
	[18.96; 23.36]	[83; 109]	[18.81; 23.42]	[103; 132]	[17.85; 21.76]	[78; 99]	[16.47; 20.55]	[91; 119]	
Imaging	2.52	91	2.87	104	2.4	89	2.87	100	0.22
	[2.25; 2.88]	[81; 107]	[2.57; 3.26]	[92; 118]	[2.14; 2.78]	[77; 106]	[2.59; 3.22]	[89; 115]	
Other	0.73	21	0.84	25	0.6	19	0.87	25	0.54
	[0.61; 0.87]	[17; 26]	[0.68; 1.01]	[20; 31]	[0.49; 0.7]	[16; 24]	[0.72; 1.07]	[21; 31]	
Transportation	1.13	31	0.22	9	0.69	40	0.49	15	0.23
	[0.35; 3.95]	[12; 79]	[0.11; 0.46]	[4; 18]	[0.32; 1.93]	[9; 191]	[0.29; 0.87]	[9; 29]	
Medications	33.76	2214	31.68	2187	30.04	2447	31.78	2137	0.65
	[31.41; 36.3]	[1,996; 2,621]	[29.65; 33.88]	[1,938; 2,573]	[28.05; 32.35]	[2,075; 3,053]	[29.81; 34.03]	[1,918; 2,553]	
Cardiovascular system	10.69	601	9.55	554	8.52	506	8.44	470	0.018
	[9.66; 11.89]	[531; 675]	[8.67; 10.56]	[495; 635]	[7.69; 9.54]	[445; 587]	[7.59; 9.36]	[412; 529]	
Digestive and metabolism	6.55	354	6.15	343	5.42	298	5.98	333	0.37
	[5.81; 7.46]	[307; 417]	[5.39; 6.97]	[293; 404]	[4.74; 6.13]	[247; 362]	[5.32; 6.8]	[275; 398]	
Nervous system	5.48	342	5.25	356	6.41	427	5.72	343	0.15
	[4.81; 6.34]	[290; 407]	[4.6; 5.98]	[301; 426]	[5.66; 7.18]	[361; 519]	[4.99; 6.61]	[287; 411]	
Anti-depressants	1.21	66	1.03	56	1.57	85	1.17	62	0.15
	[0.95; 1.52]	[53; 88]	[0.79; 1.32]	[42; 77]	[1.27; 1.96]	[67; 115]	[0.9; 1.48]	[47; 87]	
Anxiolytics	0.97	24	0.81	20	1.03	24	0.83	21	0.50
	[0.74; 1.28]	[18; 34]	[0.6; 1.06]	[15; 27]	[0.76; 1.3]	[18; 31]	[0.62; 1.06]	[16; 29]	
Hypnotics and sedatives	0.53	16	0.49	14	0.67	20	0.71	21	0.22
	[0.37; 0.72]	[12; 23]	[0.33; 0.67]	[10; 21]	[0.5; 0.86]	[15; 27]	[0.51; 0.96]	[15; 28]	
Other nervous system drugs	2.77	236	2.92	266	3.13	297	3.02	239	0.99
	[2.34; 3.27]	[193; 292]	[2.52; 3.43]	[217; 333]	[2.66; 3.77]	[235; 385]	[2.55; 3.58]	[193; 302]	
Other drugs	11.05	917	10.73	933	9.7	1217	11.63	991	0.051
	[10.15; 12.17]	[730; 1219]	[9.71; 11.94]	[717; 1,259]	[8.84; 10.85]	[880; 1,784]	[10.68; 12.69]	[816; 1,348]	
Intervention	-	1,469	-	549	-	920	-	0	-
Total without intervention	-	7,702	-	7,951	-	7,845	-	7,106	0.77
		[6,783; 9,066]		[6,864; 9,833]		[6,800; 9,273]		[6,374; 8,103]	
Total	-	9,171	-	8,500	-	8,765	-	7,106	<0.001
		[8,311; 10,632]		[7,453; 10,592]		[7,682; 10,088]		[6,347; 8,065]	


*Confidence Interval; †p-value of Kruskal-Wallis rank sum test; ‡Medical Surgical and Obstetrics; §Polyunsaturated Fatty Acids; ∥Multidomain Intervention

At 3 years, the placebo group had the lowest inpatient costs of the three groups, and particularly for psychiatric hospitalizations that were higher in the three other groups. The PFA group had significantly higher GP, cardiologist and lab test costs than others groups (p= 0.026, p= 0.018 and p=0.090, respectively). Finally, cardiovascular medication costs were higher in the PFA + MI group (p=0.018).

### Three years costs analysis

Detailed costs for every 6 months show a substantial increase in total costs for each group between 24 and 36 months of follow-up, which was mainly due to a substantial increase in hospital costs (Appendix 2. Table A2).

Table 3 presents the results of the GLM for the whole population (1,525) and show that total costs including intervention costs increased with age, number of medical conditions and the type of intervention. It was significantly higher in the PFA + MI group and the MI group. The GLM regression of total costs without intervention costs shows that only age and the number of medical conditions increased healthcare costs significantly.

**Table 3:** Multivariate analysis of total cost with and without intervention over the 3-year follow-up period (N= 1,525)

	Total costs with intervention costs			Total costs without intervention costs		
	RR*	CI†	p‡	RR*	CI†	p‡
Age						
[69; 72]	1			1		
]72; 77]	1.16	[0.96; 1.41]	0.125	1.22	[0.97; 1.52]	0.09
]77; 87]	1.3	[1.13; 1.51]	<0.001	1.39	[1.17; 1.64]	<0.001
Gender						
Male	1			1		
Female	1	[0.87; 1.16]	0.965	0.99	[0.86; 1.15]	0.91
Intervention						
Placebo	1			1		
PFA§	1.14	[0.95; 1.36]	0.15	1.08	[0.87; 1.35]	0.48
MI ∥	1.26	[1.05; 1.52]	0.016	1.11	[0.89; 1.39]	0.34
PFA§ + MI∥	1.24	[1.05; 1.48]	0.013	1.01	[0.83; 1.22]	0.99
Baseline Z score						
]−3; −0.21]	1			1		
]−0.21; 0.29]	0.95	[0.82; 1.11]	0.545	0.92	[0.78; 1.09]	0.34
]0.29; 1.89]	0.92	[0.79; 1.08]	0.317	0.91	[0.76; 1.08]	0.26
Number of medical conditions						
[0; 5]	1			1		
]5; 8]	1.55	[1.34; 1.79]	<0.001	1.62	[1.36; 1.93]	<0.001
]8; 16]	2.24	[1.9; 2.64]	<0.001	2.39	[1.94; 2.96]	<0.001


*Relattive Risk; †Confidence Interval; ‡p-value; §Polyunsaturated Fatty Acids; ∥Multidomain Intervention

### Cost-effectiveness analysis

Differences in total costs including intervention costs between the intervention groups and the placebo group were €1,237, €1,705 and €1,986 for the PFA, MI and PFA + MI groups, respectively (Appendix 3. Table A3). Changes in Z scores between the intervention groups and the control group were 0.011 for PFA, 0.079 for MI and 0.093 for PFA + MI, respectively (Appendix 3. Table A3).

**Figure 2:**
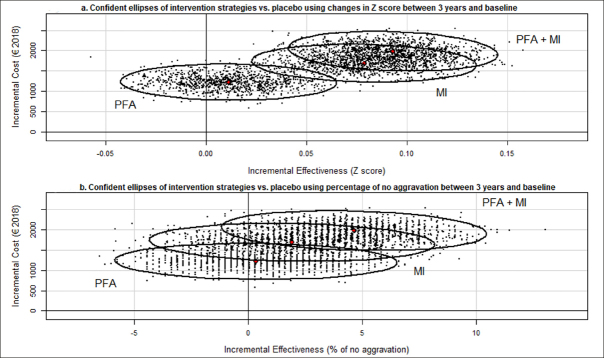
Confidence ellipses of intervention strategies versus placebo

As presented in the base case ICER scatter plot (Figure 2-a), the ICER comparing combined intervention and MI alone with placebo amounted to €21,443 and €21,543 per improved Z score point, respectively. The confidence ellipses of the ICERs comparing the PFA + MI and MI strategies overlap. All dots that represent the results of the 1,000 replications of ICERs for the PFA + MI and the MI strategies alone vs. placebo are concentrated in the northeast quadrant. As presented in the CEAC (Appendix 4. Figure A4.a), PFA + MI and MI alone have a probability of 95% to be cost-effective at a €50,000 WTP threshold. When the percentage of patients with no aggravation of cognitive functions between baseline and year 3 (Figure 2.b) is used, it can be noted that all the bootstrapped ICERs of the PFA + MI strategy vs. placebo are located in the northeast quadrant. The probability that PFA + MI and MI alone are cost-effective at a €50,000 threshold is 90% and 65%, respectively. (Appendix 4. Figure A4.b).

Results for the sensitivity analysis using the complete data set show an ICER amounting to €19,638 and €20,595 per improved Z score point for combined intervention and MI alone compared to placebo. All dots that represent the results of the 1,000 replications of ICERs for the PFA + MI and the MI strategies alone vs. placebo are concentrated in the northeast quadrant (Appendix 5).

## Discussion

This study provides first time evidence on the cost-effectiveness of preventive interventions for AD. Our study showed that PFA + MI and MI alone have an ICER of €21,443 and €21,543 respectively per improved Z score point compared to the placebo and are below the WTP of €50,000. Clinical results from the MAPT study showed that in the modified intention-to-treat population (n=1525), there were no significant differences in 3-year cognitive decline between any of the three intervention groups and the placebo group, explaining the impossibility to conclude that an intervention was most efficient than another (8). Between-group differences compared with the placebo were 0.093 (95% CI 0.001 to 0.184; adjusted p=0.142) for the combined intervention group, 0.079 (−0.012 to 0.170; adjusted p=0.179) for the MI plus placebo group, and 0.011 (−0.081 to 0.103; adjusted p=0.812) for the PFA group. Although the clinical results do not show any significant differences in efficacy between the different interventions studied, we can note a trend regarding the increase in efficacy for the combined intervention and MI groups in comparison with placebo, with a p value less than 0.2. In this context, the implementation of a cost-minimisation analysis was not appropriate, because interventions effectiveness were not strictly equivalent, that is why we choose to implement cost-effectiveness analyses. Additionally, clinical efficacy and efficiency are different measurement tools that have different aims. Efficiency measurement provides information on whether healthcare resources are used to get the best value for money while efficacy measurement determines whether an intervention produces the expected result under ideal circumstances (35).

Two efficiency studies with interventions to reduce risk factors for dementia showed cost-saving results (11, 13). The Lin et al. study used a cohort-based simultaneous equation system in United States with a lifetime time horizon. The intervention (disease management of overweight, diabetes, hypertension and other cardiovascular diseases) was cost saving (−9,259 US$ for a gain of 0.03 LY without dementia) (11). The Zhang et al. study used a Markov model in Sweden and Finland with a 20-year time horizon. The intervention (health promotion program combined with pharmacological treatment of cardiovascular risk factors) was cost saving (−21,974 SEK for a gain of 0.0511 QALY) (13). In another study on physical activity, van Baal and colleagues used a Markov model in United Kingdom with a lifetime time horizon. They calculated incremental costs of −4600 GBP to 1500 GBP depending on the scenarios (physical activity levels and adherence to recommendations), the interventions were cost saving or cost-effective depending on the context, and the maximum ICER was £2,777/LY (10). Finally, an economic evaluation of nutritional supplementation (B-vitamins) in the prevention of dementia based on stochastic decision model in United Kingdom with a lifetime time horizon was tested. Contrary to our study, the intervention was cost saving (−502 GBP for 0.008 QALY gained) (12). However, this supplementation was based on B-vitamins and not PFA. Caution should be exercised in comparing because all these studies were based on hypothetical interventions in decision models and were not RCT like our study (9).

Three years total costs amounted from €7,106 for the placebo group to €7,951 for the PFA group. Costs differences between groups were not statistically significant when interventions costs are not included and becomes significant after the inclusion of these costs. This results show that intervention costs is the main cost component, which explain the difference in total costs. Nevertheless, we can note a cost difference of at least €596 between placebo group and the other three groups. This difference is mainly lead by psychiatric hospitalizations (p=0.012). We can explain this difference by the fact that few patients are hospitalized for psychiatric reasons in each group. In the placebo group, only two psychiatric hospitalizations were found during the three years follow-up period while between four and eight psychiatric hospitalizations were found for the other groups. Moreover, we can note a significant cost difference between groups for consultation costs. This is led by the cost of general practitioner cardiologist, which were higher for PFA group compared with other groups. However, this cost difference from a clinical perspective, correspond between 0.5 to 1.5 consultations in terms of frequency during the three years follow-up period. Annualized costs amounted to €2,567; €2,650; €2,618 and €2,369, for PFA + MI, PFA alone, MI alone and placebo groups, respectively. As shown in the original clinical paper, 45% of the participants included in the MAPT study had at least one Fried frailty criterion and the other participants had none of those criteria. The sample of participants included in the MAPT study was considered as pre-frail or robust [8]. A meta-analysis published in 2019 showed that annual healthcare costs for the elderly varied from €1,217 to €2,056 for a Spanish study and from €9,193 and to €18,525 for a study performed in the USA, for robust and pre-frail older adults, respectively [36]. All the studies included in this meta-analyse took into account inpatient stays, ER and outpatient care. Total costs for Mexican and German studies, which also included formal and informal care costs, varied from €1,248 to €1,775 and from €2,568 to €3,284 for robust and pre-frail older adults, respectively (36). In a French study, the authors demonstrated that annual costs for outpatient care were €1,254 for a robust population of older adults (37). This cost was higher for participants 70–74 years of age and amounted to €1,432. In our study, the mean annualized outpatient care costs amounted to €1,315. A comparison with other studies shows that our cost results are consistent with results in published papers.

The efficacy of MI and/or PFA supplementation was estimated using a Z score. Some countries, such as the UK (NICE), recommend the use of QALY to inform decision-makers for resource allocation. We chose not to use QALY in our study because it is very limited for the elderly. Health related quality of life instruments such as EQ-5D-5L measures do not capture the maintenance of independence or the social effects of interventions, which are particularly important dimensions for the elderly [38]. The QALY metric has also been criticized for being insufficiently sensitive to measure small but clinically meaningful changes in health status or utility (39, 40). In order to provide an ICER that can be informative for clinicians and decision makers, sensitivity analyses were performed on different interpretation of effectiveness using the 0.3-point Z-score as a cut-off (25). ICERs were €434/percent of participants with no aggravation of cognitive functions between baseline and 3 years for PFA + MI compared to the placebo. The use of different interpretation of effectiveness did not change the results and confirmed that the PFA + MI strategy present an ICER under the WTP threshold.

Informal and formal care costs were not included in our analysis. Unlike for demented people for which formal and informal care costs can constitute more than 50% of total costs, these type of costs are very low in older people without dementia (41, 42). As stated in the Panaponaris et al study, only 10% of the older people without dementia needed assistance with ADL and less than 25% needed assistance with IADL (42).

Healthcare consumption was measured using ad hoc questionnaires and was based on declarative data. In order to take into account the uncertainty, we implemented probabilistic sensitivity analyses. We used Probabilistic Sensitive Analysis (PSA) instead of Determinist Sensitive Analysis (DSA) because in DSA analysis the analyst himself chooses parameters and their variation (which leads to selection bias); it only allows the simultaneous variation of a few parameters and cannot take into account the interaction between parameters (43, 44, 45, 46). Moreover, the Missing at Random characteristics of our data were verified and the issue of missing data was addressed through multiple imputation. In addition, the sensitivity analysis of the ICERs calculated with the 1,320 participants for whom economic data were available was performed and confirmed our results. Nevertheless, results are based on individual data recorded alongside an RCT, which provided us with robust data analysed using adapted statistical methods.

## Conclusions

Results show ICERS of PFA combined with MI and MI alone amounted to €21,443 and €21,543 respectively per improved Z score point compared to the placebo and are below the WTP of €50,000 while the ICER of PFA alone amounted to €111,720 per improved Z score point. These results are consolidated through the sensitivity analyses performed on different effectiveness criteria and ICER calculated using observed data on 1320 participants. The article provides additional information to strictly medical data and may serve as a basis for decision making for the FNHI and more widely for other relevant policy makers. Further results, using lifetime horizon analytical model, are necessary to complete information provided as part of this RCT based study. This study was the first to collect economic and clinical data of older people probably at risk of developing AD during a three years follow-up period. This study may help the scientific community to access additional economic data in the field of AD prevention which can be used on the one hand to build lifetime model and on the other hand, to compare results between different countries and finally contribute to improve the economic research on AD.

## Electronic supplementary material

Appendix 1 - Table A1. Sources of unit costs

Appendix 2-Table A2: Cost description according to strategies for each 6 months visits

Appendix 3- Table A3. Three-year costs, medical outcomes, effectiveness and cost-effectiveness (N=1,525)

Appendix 4- Figure A4: Acceptability curves of intervention strategies vs. placebo using different effectiveness criteria

Appendix 5 - Table A5. Three years costs, medical outcomes, effectiveness and cost-effectiveness (N=1,320)

*Funding:* The present study called ECO-MAPT study was supported by grants from the French Ministry of Health (PHRC 2008) and the France Alzheimer Association (Doctoral Scholarship 2010). The original MAPT study was supported by grants from the Toulouse Geriatric Center, the French Ministry of Health (PHRC 2008, 2009), Pierre Fabre Research Institute (manufacturer of the omega-3 supplement), ExonHit Therapeutics SA, and Avid Radiopharmaceuticals Inc. Trial registration number: NCT00672685, first registered in May 6, 2008

All authors meet criteria for authorship as stated in the COI form, as well as their contributions to the manuscript. All authors' specific areas of contributions is listed, using categories below: — Study concept and design: Nadège Costa, Hélène Derumeaux, Sophie Guyonnet, Isabelle Carrié, Sandrine Andrieu, Laurent Molinier; — Acquisition of data: Nadège Costa, Hélène Derumeaux, Sophie Guyonnet, Isabelle Carrié, Sandrine Andrieu, Laurent Molinier; — Analysis and interpretation of data: Nadège Costa, Michael Mounié, Arnaud Pagès, Hélène Derumeaux, Nicola Coley, Chrsitelle Cantet, Sandrine Andrieu, Laurent Molinier; — Drafting of the manuscript: Nadège Costa, Michael Mounié, Arnaud Pagès, Hélène Derumeaux, Laurent Molinier; — Critical revision of the manuscript for important intellectual content: Thomas Rapp, Nicola Coley, Sandrine Andrieu.

*Ethics approval and consent to participate:* The investigating physicians, who verified inclusion and exclusion criteria and obtained written informed consent, recruited all participants. The trial protocol was approved by the French Ethical Committee located in Toulouse (CPP SOOM II) and was authorised by the French Health Authority.
