# Supplementary material for: The Cost-Effectiveness of Three Prevention Strategies in Alzheimer's Disease: Results from the Multidomain Alzheimer Preventive Trial (MAPT)
Source: J Prev Alzheimers Dis. 2021 Aug 2;8(4):425–35. doi: 10.14283/jpad.2021.47 (PMC12280784; doi:10.14283/jpad.2021.47)
Supplement: Supplementary file 1 — Appendix 1 - Table A1. Sources of unit costs [file mmc1.docx]

**Appendix 1 – Table A1. Sources of unit costs**

| **Cost components** | **Unit** | **Cost per unit (€, 2018)** | **Sources** |
| --- | --- | --- | --- |
| **Participant stays (Exact value)** |  |  |  |
| *Hospitalization* | Participant stay | From 2,112 to 5,912 | French Technical Agency for Medical Information on Hospitalization |
| *Day Hospitalization* | Day | From 516 to 1,865 | French Technical Agency for Medical Information on Hospitalization |
| *Rehabilitation* | Day | 574 | University hospital of Toulouse |
| *Psychiatry* | Day | 771 | University hospital of Toulouse |
| **Emergency room (Exact value)** | Visit | 190 | University hospital of Toulouse |
| **Consultation (Exact value)** |  |  |  |
| *General practitioner* | Visit | 16.5 | French Healthcare Insurance (GNPA*) |
| *Cardiologist* | Visit | 32,411 | French Healthcare Insurance (GNPA*) |
| *Neurologist/Psychiatrist* | Visit | 26.3 | French Healthcare Insurance (GNPA*) |
| *Other specialists* | Visit | 16.5 | French Healthcare Insurance |
| **Medical procedures (Mean [Min-Max]** |  |  |  |
| *Imaging* | Exam | 39.37 [13.96 - 782.00] | French Healthcare Insurance (CCMA^†^) |
| *Biology* | Exam | 6.49 [0.65 - 752.98] | French Healthcare Insurance (NMBA^‡^) |
| *Other medical procedures* | Exam | 32.64 [6.72 - 204.36] | French Healthcare Insurance (CCMA^†^) |
| **Paramedical procedures (Mean Γ(shape; scale))** |  |  |  |
| *Nurse* | Visit | 22.10 Γ(0.70; 31.63) | French Healthcare Insurance (GSB^§^) |
| *Physiotherapist* | Visit | 19.48 Γ(7.75; 2.51) | French Healthcare Insurance (GSB^§^) |
| ***Medication (Exact value/daily dose)*** | Daily dose | From 0.04 to 210 | French Healthcare Insurance |
| **Transportation (Mean Γ(shape; scale))** |  |  |  |
| *Ambulance* | Trip | 80.25 Γ(2.43; 33.06) | French Healthcare Insurance database (GSB^§^) |
| *NEMT^\|\|^* | Trip | 33 Γ(1.4; 23.25) | French Healthcare Insurance database (GSB^§^) |
| *Taxi* | Trip | 51.85 Γ(0.89; 58.49) | French Healthcare Insurance database (GSB^§^) |

**GNPA: General Nomenclature of Professional Acts;* †*CCMA: Common Classification of Medical Acts;* ‡*NMBA: Nomenclature of Medical Biological Acts;* §*GSB: Generalist Sample of the Beneficiaries; ||NEMT: Non-emergency medical transportation*
