# Supplementary material for: The Cost-Effectiveness of Three Prevention Strategies in Alzheimer's Disease: Results from the Multidomain Alzheimer Preventive Trial (MAPT)
Source: J Prev Alzheimers Dis. 2021 Aug 2;8(4):425–35. doi: 10.14283/jpad.2021.47 (PMC12280784; doi:10.14283/jpad.2021.47)
Supplement: Supplementary file 2 — Appendix 2-Table A2: Cost description according to strategies for each 6 months visits [file mmc2.docx]

**Appendix 2-Table A2: Cost description according to strategies for each 6 months visits**

Appendix2.1: Cost (€2018) description according to the cost component and strategies at visit no. 1 (n=1,320)

|  | | | **PFA**^†^ **+ MI*** | | **PFA**^†^ | | **MI*** | | **Control** | |  |
| --- | --- | --- | --- | --- | --- | --- | --- | --- | --- | --- | --- |
|  |  |  | Frequency | Cost | Frequency | Cost | Frequency | Cost | Frequency | Cost | p^§^ |
|  |  |  | Mean  [95% CI^‡^] | Mean  [95% CI^‡^] | Mean  [95% CI^‡^] | Mean  [95% CI^‡^] | Mean  [95% CI^‡^] | Mean  [95% CI^‡^] | Mean  [95% CI^‡^] | Mean  [95% CI^‡^] |  |
| **Inpatient stays** | | | 0.14  [0.09; 0.18] | 509  [328; 796] | 0.23  [0.16; 0.45] | 724  [471; 1407] | 0.14  [0.1; 0.2] | 402  [272; 586] | 0.17  [0.13; 0.22] | 578  [415; 831] | 0.42 |
| MSO^\|\|^ <1 day | | 0.05  [0.02; 0.07] | 48  [24; 79] | 0.12  [0.06; 0.3] | 81  [48; 126] | 0.05  [0.03; 0.08] | 52  [31; 82] | 0.06  [0.04; 0.09] | 66  [41; 103] | 0.51 |  |
| MSO^\|\|^  ≥1 day | | 0.08  [0.06; 0.12] | 385  [243; 577] | 0.09  [0.06; 0.14] | 396  [264; 590] | 0.09  [0.06; 0.14] | 350  [235; 537] | 0.1  [0.07; 0.13] | 445  [311; 652] | 0.79 |  |
| Post-acute & rehabilitation care unit | | 0.01  [0; 0.02] | 77  [0; 226] | 0.01  [0; 0.02] | 116  [0; 507] | 0  [0; 0] | 0  [0; 0] | 0.01  [0; 0.02] | 67  [0; 204] | 0.57 |  |
| Psychiatric unit care | | 0  [0; 0] | 0  [0; 0] | 0  [0; 0.01] | 132  [0; 396] | 0  [0; 0] | 0  [0; 0] | 0  [0; 0] | 0  [0; 0] | 0.39 |  |
| **Emergency** | | | 0.01  [0; 0.02] | 1  [0; 3] | 0.02  [0; 0.03] | 3  [1; 6] | 0.01  [0; 0.02] | 1  [0; 3] | 0  [0; 0.01] | 1  [0; 2] | 0.29 |
| **Consultation** | | | 4.25  [3.96; 4.67] | 75  [70; 82] | 4.92  [4.52; 5.43] | 88  [81; 99] | 4.37  [3.98; 4.88] | 78  [70; 88] | 4.44  [4.07; 4.85] | 79  [73; 87] | 0.033 |
| General practitioner | | 2.52  [2.31; 2.75] | 42  [38; 46] | 2.82  [2.6; 3.08] | 47  [43; 51] | 2.46  [2.26; 2.68] | 41  [37; 44] | 2.55  [2.36; 2.8] | 42  [39; 46] | 0.10 |  |
| Specialist | | 1.73  [1.52; 1.97] | 34  [29; 39] | 2.1  [1.81; 2.54] | 42  [36; 52] | 1.92  [1.6; 2.3] | 37  [31; 45] | 1.89  [1.63; 2.2] | 37  [32; 43] | 0.34 |  |
| Cardiologist | 0.27  [0.21; 0.38] | 9  [6; 12] | 0.34  [0.27; 0.43] | 11  [9; 14] | 0.25  [0.2; 0.31] | 8  [6; 10] | 0.3  [0.23; 0.38] | 10  [8; 12] | 0.38 |  |  |
| Psychiatrist & Neurologist | 0.08  [0.04; 0.15] | 2  [1; 4] | 0.17  [0.07; 0.49] | 4  [2; 13] | 0.14  [0.06; 0.31] | 4  [2; 9] | 0.12  [0.04; 0.32] | 3  [1; 10] | 0.96 |  |  |
| Rheumatologist | 0.26  [0.19; 0.35] | 4  [3; 6] | 0.41  [0.31; 0.53] | 7  [5; 9] | 0.36  [0.27; 0.48] | 6  [4; 8] | 0.23  [0.16; 0.3] | 4  [3; 5] | 0.08 |  |  |
| Ophthalmologist | 0.31  [0.23; 0.41] | 5  [4; 7] | 0.26  [0.2; 0.36] | 4  [3; 6] | 0.29  [0.21; 0.39] | 5  [4; 6] | 0.38  [0.28; 0.49] | 6  [5; 8] | 0.39 |  |  |
| Other specialists | 0.81  [0.67; 0.98] | 13  [11; 16] | 0.92  [0.74; 1.28] | 15  [12; 21] | 0.87  [0.69; 1.18] | 14  [11; 20] | 0.87  [0.73; 1.09] | 14  [12; 18] | 0.71 |  |  |
| **Paramedical procedures** | | | 3.54  [2.63; 4.81] | 75  [53; 112] | 6.51  [3.8; 16.71] | 137  [67; 533] | 4.11  [3.08; 5.94] | 82  [57; 145] | 4.54  [3.58; 5.91] | 72  [55; 94] | 0.34 |
| Nurse | | 0.31  [0.19; 0.56] | 11  [5; 27] | 1.66  [0.46; 7.19] | 69  [7; 307] | 0.46  [0.2; 1.4] | 20  [4; 103] | 0.7  [0.41; 1.19] | 9  [5; 20] | 0.71 |  |
| Physiotherapist | | 2.92  [2.05; 4.08] | 63  [43; 94] | 3.19  [2.26; 4.61] | 64  [46; 101] | 3.2  [2.34; 4.25] | 61  [45; 89] | 3.13  [2.4; 4.13] | 61  [45; 80] | 0.52 |  |
| Other | | 0.31  [0.18; 0.53] | 1  [0; 1] | 1.66  [0.44; 6.38] | 4  [1; 16] | 0.46  [0.18; 1.23] | 1  [1; 3] | 0.7  [0.37; 1.19] | 2  [1; 3] | 0.69 |  |
| **Medical procedures** | | | 3.91  [3.3; 4.65] | 34  [28; 41] | 4.3  [3.73; 4.95] | 39  [34; 46] | 3.55  [3.04; 4.14] | 31  [27; 37] | 3.84  [3.32; 4.56] | 35  [31; 41] | 0.21 |
| Laboratory tests | | 3.37  [2.88; 4] | 14  [11; 17] | 3.63  [3.08; 4.25] | 16  [13; 20] | 3.02  [2.53; 3.53] | 13  [11; 15] | 3.22  [2.7; 3.8] | 13  [11; 15] | 0.28 |  |
| Imaging | | 0.45  [0.36; 0.58] | 17  [14; 25] | 0.52  [0.42; 0.63] | 19  [15; 25] | 0.42  [0.35; 0.51] | 16  [13; 19] | 0.49  [0.41; 0.58] | 19  [15; 23] | 0.29 |  |
| Other | | 0.09  [0.06; 0.14] | 2  [1; 4] | 0.14  [0.1; 0.2] | 4  [3; 6] | 0.11  [0.07; 0.15] | 3  [2; 5] | 0.14  [0.09; 0.19] | 4  [2; 6] | 0.39 |  |
| **Transportation** | | | 0.1  [0.03; 0.43] | 1  [0; 4] | 0.01  [0; 0.02] | 0  [0; 0] | 0.05  [0.01; 0.17] | 2  [0; 8] | 0.09  [0.02; 0.22] | 3  [1; 6] | 0.42 |
| **Medications** | | | 5.21  [4.85; 5.66] | 317  [282; 385] | 5.02  [4.67; 5.41] | 353  [303; 446] | 4.69  [4.31; 5.12] | 419  [316; 752] | 4.92  [4.58; 5.28] | 332  [291; 410] | 0.68 |
| Cardiovascular system | | 1.7  [1.51; 1.91] | 91  [81; 104] | 1.58  [1.42; 1.74] | 90  [80; 104] | 1.35  [1.19; 1.5] | 77  [67; 88] | 1.39  [1.23; 1.55] | 76  [67; 87] | 0.07 |  |
| GI** tract and metabolism | | 0.99  [0.86; 1.12] | 51  [43; 62] | 0.94  [0.82; 1.09] | 51  [43; 60] | 0.8  [0.69; 0.93] | 44  [36; 54] | 0.9  [0.79; 1.02] | 51  [42; 63] | 0.42 |  |
| Nervous system | | 1.71  [1.53; 1.9] | 127  [98; 194] | 1.69  [1.51; 1.89] | 157  [114; 246] | 1.5  [1.32; 1.7] | 232  [134; 559] | 1.79  [1.63; 1.97] | 155  [122; 221] | 0.044 |  |
| Anti-depressants | 0.82  [0.69; 0.94] | 48  [39; 59] | 0.81  [0.69; 0.94] | 55  [44; 67] | 1.05  [0.91; 1.2] | 65  [53; 80] | 0.84  [0.73; 0.97] | 50  [42; 60] | 0.16 |  |  |
| Anxiolytics | 0.19  [0.14; 0.25] | 10  [7; 13] | 0.16  [0.11; 0.21] | 8  [6; 11] | 0.25  [0.2; 0.32] | 14  [10; 22] | 0.17  [0.13; 0.22] | 10  [7; 14] | 0.17 |  |  |
| Hypnotics and sedatives | 0.15  [0.1; 0.19] | 3  [3; 5] | 0.13  [0.09; 0.17] | 3  [2; 4] | 0.17  [0.12; 0.22] | 4  [3; 5] | 0.13  [0.09; 0.17] | 3  [2; 5] | 0.67 |  |  |
| Other nervous system  drugs | 0.39  [0.32; 0.47] | 2  [2; 3] | 0.44  [0.36; 0.52] | 2  [2; 4] | 0.5  [0.4; 0.6] | 4  [3; 5] | 0.43  [0.35; 0.51] | 3  [2; 5] | 0.06 |  |  |
| Other drugs | | 0.08  [0.06; 0.12] | 32  [26; 43] | 0.08  [0.05; 0.11] | 41  [33; 53] | 0.13  [0.09; 0.16] | 44  [34; 57] | 0.11  [0.08; 0.15] | 33  [26; 42] | 0.96 |  |
| **Intervention** | | | - | 311.5 | - | 91.5 | - | 220 | - | 0 | - |
| **Total without intervention** | | | - | 1,013  [828; 1310] | - | 1,345  [1,031; 1,909] | - | 1,015  [824; 1454] | - | 1,100  [928; 1,354] | 0.32 |
| **Total** | | | - | 1,324  [1,141; 1635] | - | 1,436  [1,096; 2,004] | - | 1,235  [1,037; 1,586] | - | 1,100  [918; 1,382] | <0.001 |

* Multidomain Intervention; †Polyunsaturated Fatty Acids; ‡Confidence Interval; §p-value of Kruskal-Wallis rank sum test; || Medical Surgery Obstetrics; **GastroIntestinal

Appendix 2.2: Cost (€2018) description according to the cost component and strategies at visit no. 2 (n=1,320)

|  | | | **PFA**^†^ **+ MI*** | | **PFA**^†^ | | **MI*** | | **Control** | |  |
| --- | --- | --- | --- | --- | --- | --- | --- | --- | --- | --- | --- |
|  |  |  | Frequency | Cost | Frequency | Cost | Frequency | Cost | Frequency | Cost | p^§^ |
|  |  |  | Mean  [95% CI^‡^] | Mean  [95% CI^‡^] | Mean  [95% CI^‡^] | Mean  [95% CI^‡^] | Mean  [95% CI^‡^] | Mean  [95% CI^‡^] | Mean  [95% CI^‡^] | Mean  [95% CI^‡^] |  |
| **Inpatient stays** | | | 0.15  [0.1; 0.19] | 461  [322; 662] | 0.21  [0.15; 0.32] | 630  [434; 989] | 0.16  [0.11; 0.22] | 729  [454; 1227] | 0.16  [0.12; 0.22] | [364; 863] | 0.79 |
| MSO^\|\|^ <1 day | | 0.05  [0.02; 0.07] | 47  [25; 83] | 0.11  [0.07; 0.25] | 113  [70; 192] | 0.06  [0.03; 0.09] | 60  [35; 94] | 0.06  [0.04; 0.1] | 61  [36; 103] | 0.30 |  |
| MSO^\|\|^ ≥1 day | | 0.09  [0.06; 0.13] | 376  [246; 561] | 0.09  [0.05; 0.12] | 389  [253; 587] | 0.09  [0.06; 0.13] | 394  [246; 606] | 0.09  [0.06; 0.13] | 399  [247; 569] | 0.89 |  |
| Post-acute & rehabilitation care unit | | 0  [0; 0.01] | 30  [0; 89] | 0.01  [0; 0.03] | 128  [0; 428] | 0  [0; 0.01] | 50  [0; 151] | 0.01  [0; 0.02] | 98  [0; 296] | 0.88 |  |
| Psychiatric unit care | | 0  [0; 0.01] | 9  [0; 28] | 0  [0; 0] | 0  [0; 0] | 0.01  [0; 0.02] | 224  [44; 664] | 0  [0; 0] | 0  [0; 0] | 0.11 |  |
| **Emergency** | | | 0.02  [0; 0.03] | 3  [1; 6] | 0.01  [0; 0.02] | 1  [0; 3] | 0.01  [0; 0.02] | 2  [1; 5] | 0  [0; 0.01] | 1  [0; 2] | 0.34 |
| **Consultation** | | | 4.29  [3.96; 4.65] | 76  [71; 83] | 4.66  [4.29; 5.07] | 83  [76; 91] | 4.34  [3.98; 4.77] | 76  [69; 84] | 4.69  [4.31; 5.19] | 83  [75; 93] | 0.33 |
| General practitioner | | 2.58  [2.38; 2.83] | 43  [39; 46] | 2.77  [2.53; 3.05] | 46  [42; 50] | 2.57  [2.37; 2.83] | 42  [39; 47] | 2.49  [2.3; 2.7] | 41  [38; 45] | 0.45 |  |
| Specialist | | 1.71  [1.49; 1.93] | 34  [30; 39] | 1.89  [1.65; 2.21] | 37  [33; 45] | 1.77  [1.53; 2.06] | 33  [29; 39] | 2.21  [1.87; 2.59] | 42  [36; 51] | 0.22 |  |
| Cardiologist | 0.32  [0.26; 0.4] | 10  [8; 13] | 0.33  [0.26; 0.4] | 11  [9; 13] | 0.21  [0.16; 0.3] | 7  [5; 10] | 0.29  [0.23; 0.37] | 9  [7; 12] | 0.010 |  |  |
| Psychiatrist & Neurologist | 0.05  [0.02; 0.09] | 1  [1; 2] | 0.08  [0.03; 0.22] | 2  [1; 7] | 0.09  [0.03; 0.18] | 2  [1; 5] | 0.14  [0.05; 0.51] | 4  [1; 13] | 0.87 |  |  |
| Rheumatologist | 0.3  [0.22; 0.39] | 5  [4; 7] | 0.39  [0.29; 0.52] | 6  [5; 9] | 0.4  [0.28; 0.57] | 7  [5; 10] | 0.27  [0.2; 0.37] | 5  [3; 6] | 0.53 |  |  |
| Ophthalmologist | 0.29  [0.23; 0.37] | 5  [4; 6] | 0.32  [0.24; 0.41] | 5  [4; 7] | 0.29  [0.22; 0.42] | 5  [4; 7] | 0.38  [0.29; 0.49] | 6  [5; 8] | 0.75 |  |  |
| Other specialists | 0.75  [0.61; 0.93] | 12  [10; 16] | 0.79  [0.66; 0.96] | 13  [11; 16] | 0.78  [0.64; 0.94] | 13  [10; 16] | 1.13  [0.92; 1.58] | 19  [15; 24] | 0.40 |  |  |
| **Paramedical procedures** | | | 4.49  [2.99; 8.37] | 115  [63; 276] | 6.94  [3.44; 23] | 178  [72; 676] | 3.71  [2.7; 6.03] | 60  [43; 88] | 2.95  [2.36; 3.87] | 54  [42; 73] | 0.25 |
| Nurse | | 1.12  [0.5; 3.12] | 64  [15; 285] | 2.02  [0.41; 8.26] | 107  [8; 570] | 0.54  [0.2; 1.82] | 5  [2; 11] | 0.15  [0.08; 0.28] | 3  [1; 8] | 0.55 |  |
| Physiotherapist | | 2.25  [1.56; 3.08] | 48  [34; 73] | 2.89  [2.08; 4.16] | 65  [46; 95] | 2.63  [1.84; 3.72] | 53  [38; 80] | 2.66  [2.01; 3.48] | 51  [39; 73] | 0.21 |  |
| Other | | 1.12  [0.5; 3.1] | 3  [1; 9] | 2.02  [0.4; 8.5] | 5  [1; 31] | 0.54  [0.21; 1.71] | 1  [1; 4] | 0.15  [0.08; 0.28] | 0  [0; 1] | 0.56 |  |
| **Medical procedures** | | | 4.28  [3.6; 5.42] | 38  [32; 48] | 4.17  [3.54; 5.1] | 36  [31; 43] | 3.18  [2.72; 3.75] | 27  [23; 33] | 3.47  [2.94; 4.09] | 34  [29; 40] | 0.23 |
| Laboratory tests | | 3.7  [3.05; 4.79] | 17  [13; 26] | 3.58  [2.97; 4.4] | 16  [13; 20] | 2.7  [2.26; 3.26] | 10  [9; 13] | 2.87  [2.37; 3.52] | 13  [11; 17] | 0.22 |  |
| Imaging | | 0.43  [0.35; 0.52] | 17  [13; 22] | 0.45  [0.36; 0.55] | 16  [13; 19] | 0.36  [0.29; 0.44] | 13  [10; 17] | 0.43  [0.36; 0.53] | 15  [12; 20] | 0.50 |  |
| Other | | 0.15  [0.11; 0.22] | 4  [3; 6] | 0.14  [0.1; 0.19] | 5  [3; 7] | 0.12  [0.08; 0.17] | 4  [3; 6] | 0.17  [0.12; 0.23] | 5  [4; 7] | 0.71 |  |
| **Transportation** | | | 0.2  [0.03; 0.82] | 2  [1; 7] | 0.02  [0; 0.06] | 1  [0; 2] | 0.01  [0; 0.03] | 0  [0; 2] | 0.04  [0.02; 0.09] | 1  [1; 3] | 0.161 |
| **Medications** | | | 5.41  [5; 5.84] | 336  [295; 405] | 5.08  [4.71; 5.43] | 412  [324; 603] | 4.77  [4.36; 5.2] | 355  [299; 448] | 5.14  [4.76; 5.49] | 347  [303; 428] | 0.49 |
| Cardiovascular system | | 1.76  [1.55; 1.97] | 96  [84; 111] | 1.58  [1.42; 1.75] | 88  [78; 102] | 1.35  [1.2; 1.53] | 75  [65; 86] | 1.42  [1.25; 1.59] | 79  [69; 90] | 0.046 |  |
| GI** tract and metabolism | | 1.06  [0.93; 1.22] | 55  [46; 66] | 0.97  [0.84; 1.11] | 50  [42; 59] | 0.85  [0.74; 0.99] | 44  [36; 54] | 0.92  [0.8; 1.06] | 53  [44; 66] | 0.37 |  |
| Nervous system | | 1.72  [1.56; 1.89] | 130  [102; 196] | 1.72  [1.53; 1.92] | 221  [132; 390] | 1.53  [1.36; 1.73] | 167  [118; 268] | 1.86  [1.7; 2.03] | 160  [124; 231] | 0.019 |  |
| Anti-depressants | 0.87  [0.74; 1] | 55  [44; 69] | 0.8  [0.69; 0.93] | 53  [43; 65] | 1.03  [0.9; 1.18] | 69  [57; 85] | 0.95  [0.8; 1.1] | 55  [45; 66] | 0.12 |  |  |
| Anxiolytics | 0.2  [0.15; 0.25] | 12  [9; 18] | 0.17  [0.12; 0.22] | 9  [7; 12] | 0.26  [0.2; 0.33] | 15  [11; 20] | 0.2  [0.15; 0.25] | 10  [8; 14] | 0.17 |  |  |
| Hypnotics and sedatives | 0.16  [0.12; 0.22] | 5  [3; 8] | 0.12  [0.08; 0.16] | 3  [2; 4] | 0.16  [0.12; 0.22] | 4  [3; 5] | 0.14  [0.1; 0.18] | 3  [2; 5] | 0.65 |  |  |
| Other nervous system  drugs | 0.42  [0.35; 0.51] | 3  [2; 4] | 0.45  [0.37; 0.54] | 2  [1; 3] | 0.49  [0.4; 0.6] | 4  [3; 5] | 0.5  [0.41; 0.59] | 4  [3; 5] | 0.08 |  |  |
| Other drugs | | 0.09  [0.06; 0.12] | 36  [28; 46] | 0.07  [0.04; 0.1] | 39  [30; 51] | 0.12  [0.08; 0.15] | 47  [37; 60] | 0.12  [0.08; 0.16] | 37  [30; 47] | 0.91 |  |
| **Intervention** | | | - | 311.5 | - | 91.5 | - | 220 | - | 0 | - |
| **Total without intervention** | | | - | 1032  [860; 1,260] | - | 1341  [1,041; 1,829] | - | 1,250  [962; 1,737] | - | 1,080  [878; 1,379] | 0.19 |
| **Total** | | | - | 1343  [1,175; 1,560] | - | 1432  [1,133; 2,006] | - | 1470  [1,199; 2,017] | - | 1080  [880; 1,378] | <0.001 |

* Multidomain Intervention; †Polyunsaturated Fatty Acids; ‡Confidence Interval; §p-value of Kruskal-Wallis rank sum test; || Medical Surgery Obstetrics; **GastroIntestinal

Appendix 2.3: Cost (€2018) description according to the cost component and strategies at visit no. 3 (n=1,320)

|  | | | **PFA**^†^ **+ MI*** | | **PFA**^†^ | | **MI*** | | **Control** | |  |
| --- | --- | --- | --- | --- | --- | --- | --- | --- | --- | --- | --- |
|  |  |  | Frequency | Cost | Frequency | Cost | Frequency | Cost | Frequency | Cost | p^§^ |
|  |  |  | Mean  [95% CI^‡^] | Mean  [95% CI^‡^] | Mean  [95% CI^‡^] | Mean  [95% CI^‡^] | Mean  [95% CI^‡^] | Mean  [95% CI^‡^] | Mean  [95% CI^‡^] | Mean  [95% CI^‡^] |  |
| **Inpatient stays** | | | 0.15  [0.11; 0.21] | 587  [374; 950] | 0.16  [0.11; 0.21] | 542  [356; 837] | 0.17  [0.12; 0.22] | 568  [409; 812] | 0.13  [0.09; 0.18] | 510  [322; 921] | 0.41 |
| MSO^\|\|\|^ <1 day | | 0.05  [0.02; 0.08] | 48  [24; 85] | 0.06  [0.03; 0.08] | 57  [33; 99] | 0.05  [0.02; 0.07] | 44  [25; 70] | 0.05  [0.02; 0.07] | 48  [24; 85] | 0.90 |  |
| MSO^\|\|^ ≥1 day | | 0.1  [0.06; 0.15] | 425  [287; 649] | 0.09  [0.06; 0.13] | 366  [250; 550] | 0.12  [0.08; 0.18] | 525  [356; 749] | 0.07  [0.04; 0.1] | 315  [199; 482] | 0.52 |  |
| Post-acute & rehabilitation care unit | | 0.01  [0; 0.02] | 113  [0; 419] | 0.01  [0; 0.02] | 51  [0; 214] | 0  [0; 0] | 0  [0; 0] | 0.01  [0; 0.03] | 79  [0; 360] | 0.57 |  |
| Psychiatric unit care | | 0  [0; 0] | 0  [0; 0] | 0  [0; 0.01] | 68  [0; 205] | 0  [0; 0] | 0  [0; 0] | 0  [0; 0.01] | 67  [0; 201] | 0.57 |  |
| **Emergency** | | | 0.02  [0; 0.04] | 3  [1; 7] | 0.01  [0; 0.02] | 2  [0; 4] | 0  [0; 0.01] | 1  [0; 2] | 0.02  [0; 0.03] | 3  [1; 7] | 0.57 |
| **Consultation** | | | 4.14  [3.8; 4.56] | 74  [68; 82] | 4.74  [4.37; 5.15] | 84  [77; 91] | 4.22  [3.84; 4.61] | 76  [69; 83] | 4.35  [3.95; 4.8] | 76  [69; 84] | 0.048 |
| General practitioner | | 2.45  [2.22; 2.71] | 40  [37; 45] | 2.77  [2.56; 3.03] | 46  [42; 50] | 2.26  [2.09; 2.44] | 37  [34; 40] | 2.36  [2.18; 2.6] | 39  [36; 42] | 0.002 |  |
| Specialist | | 1.69  [1.46; 1.95] | 34  [29; 39] | 1.98  [1.71; 2.3] | 38  [33; 44] | 1.96  [1.71; 2.32] | 38  [33; 45] | 1.98  [1.7; 2.38] | 37  [33; 44] | 0.85 |  |
| Cardiologist | 0.34  [0.28; 0.42] | 11  [9; 13] | 0.3  [0.23; 0.37] | 10  [7; 12] | 0.3  [0.23; 0.38] | 10  [8; 13] | 0.22  [0.16; 0.29] | 7  [5; 9] | 0.027 |  |  |
| Psychiatrist & Neurologist | 0.06  [0.03; 0.1] | 2  [1; 3] | 0.06  [0.02; 0.17] | 2  [1; 4] | 0.13  [0.08; 0.22] | 4  [2; 6] | 0.1  [0.05; 0.2] | 3  [1; 6] | 0.20 |  |  |
| Rheumatologist | 0.3  [0.22; 0.42] | 5  [4; 7] | 0.29  [0.21; 0.38] | 5  [3; 6] | 0.35  [0.25; 0.61] | 6  [4; 10] | 0.28  [0.2; 0.37] | 5  [3; 6] | 0.10 |  |  |
| Ophthalmologist | 0.31  [0.23; 0.42] | 5  [4; 7] | 0.37  [0.29; 0.5] | 6  [5; 8] | 0.37  [0.28; 0.51] | 6  [5; 8] | 0.33  [0.25; 0.43] | 5  [4; 7] | 0.71 |  |  |
| Other specialists | 0.68  [0.55; 0.86] | 11  [9; 14] | 0.95  [0.77; 1.25] | 16  [13; 19] | 0.8  [0.66; 0.97] | 13  [11; 16] | 1.06  [0.86; 1.43] | 17  [14; 23] | 0.32 |  |  |
| **Paramedical procedures** | | | 4.07  [2.61; 9.12] | 58  [42; 90] | 3.46  [2.52; 5.02] | 57  [41; 84] | 3.38  [2.34; 4.69] | 47  [34; 70] | 2.92  [2.15; 3.97] | 55  [39; 78] | 0.51 |
| Nurse | | 0.89  [0.27; 3.25] | 14  [6; 45] | 0.43  [0.17; 1.17] | 11  [3; 34] | 0.77  [0.37; 1.38] | 9  [4; 21] | 0.3  [0.14; 0.69] | 9  [3; 35] | 0.35 |  |
| Physiotherapist | | 2.28  [1.62; 3.18] | 42  [29; 60] | 2.61  [1.86; 3.49] | 45  [32; 63] | 1.84  [1.2; 2.72] | 36  [24; 59] | 2.31  [1.69; 3.33] | 45  [31; 67] | 0.12 |  |
| Other | | 0.89  [0.25; 3.18] | 2  [1; 9] | 0.43  [0.17; 1.32] | 1  [0; 3] | 0.77  [0.41; 1.47] | 2  [1; 4] | 0.3  [0.14; 0.68] | 1  [0; 2] | 0.34 |  |
| **Medical procedures** | | | 4.31  [3.53; 5.04] | 36  [31; 43] | 3.79  [3.3; 4.43] | 35  [29; 42] | 4.24  [3.56; 4.98] | 39  [31; 59] | 3.26  [2.8; 3.83] | 38  [32; 48] | 0.87 |
| Laboratory tests | | 3.7  [3.09; 4.48] | 15  [13; 18] | 3.26  [2.71; 3.82] | 16  [13; 20] | 3.64  [3.06; 4.34] | 14  [12; 17] | 2.66  [2.22; 3.12] | 17  [14; 22] | 0.99 |  |
| Imaging | | 0.47  [0.38; 0.57] | 17  [14; 21] | 0.41  [0.32; 0.51] | 15  [11; 20] | 0.46  [0.34; 0.74] | 20  [14; 43] | 0.46  [0.36; 0.57] | 16  [12; 21] | 0.80 |  |
| Other | | 0.14  [0.09; 0.22] | 4  [2; 7] | 0.13  [0.09; 0.19] | 4  [3; 7] | 0.14  [0.1; 0.19] | 5  [3; 7] | 0.14  [0.09; 0.22] | 5  [3; 8] | 0.81 |  |
| **Transportation** | | | 0.02  [0; 0.07] | 0  [0; 1] | 0.04  [0; 0.16] | 2  [0; 6] | 0.04  [0.01; 0.08] | 2  [0; 6] | 0.05  [0.02; 0.09] | 1  [1; 3] | 0.43 |
| **Medications** | | | 5.53  [5.11; 5.98] | 334  [295; 393] | 5.2  [4.85; 5.62] | 355  [307; 431] | 5.05  [4.67; 5.44] | 418  [340; 575] | 5.14  [4.77; 5.55] | 342  [299; 404] | 0.83 |
| Cardiovascular system | | 1.71  [1.52; 1.91] | 94  [82; 107] | 1.58  [1.41; 1.74] | 96  [84; 110] | 1.42  [1.27; 1.57] | 79  [68; 91] | 1.38  [1.24; 1.55] | 77  [67; 89] | 0.043 |  |
| GI** tract and metabolism | | 1.07  [0.93; 1.22] | 52  [44; 62] | 1  [0.88; 1.14] | 55  [47; 66] | 0.91  [0.79; 1.04] | 50  [41; 60] | 0.97  [0.84; 1.11] | 55  [45; 68] | 0.69 |  |
| Nervous system | | 1.83  [1.64; 2.03] | 135  [105; 196] | 1.77  [1.58; 2.01] | 145  [111; 226] | 1.65  [1.48; 1.85] | 220  [146; 376] | 1.85  [1.68; 2.05] | 154  [124; 217] | 0.37 |  |
| Anti-depressants | 0.93  [0.79; 1.1] | 52  [44; 62] | 0.86  [0.74; 0.99] | 58  [49; 74] | 1.07  [0.94; 1.22] | 68  [57; 84] | 0.94  [0.81; 1.1] | 55  [46; 68] | 0.16 |  |  |
| Anxiolytics | 0.19  [0.15; 0.25] | 10  [7; 13] | 0.17  [0.13; 0.23] | 10  [7; 13] | 0.27  [0.21; 0.33] | 14  [10; 18] | 0.22  [0.17; 0.29] | 11  [8; 14] | 0.14 |  |  |
| Hypnotics and sedatives | 0.17  [0.12; 0.22] | 4  [3; 5] | 0.14  [0.1; 0.18] | 3  [3; 5] | 0.18  [0.14; 0.23] | 4  [3; 6] | 0.14  [0.1; 0.17] | 4  [3; 5] | 0.55 |  |  |
| Other nervous system  drugs | 0.49  [0.4; 0.59] | 2  [2; 3] | 0.47  [0.39; 0.56] | 3  [2; 4] | 0.51  [042; 0,62] | 3  [2; 4] | 0,46  [0,38; 0,55] | 4  [3; 5] | 0,19 |  |  |
| Other drugs | | 0,08  [0,06; 0,12] | 36  [29; 44] | 0,08  [0,05; 0,11] | 43  [33; 54] | 0,11  [0,08; 0,15] | 48  [37; 63] | 0,12  [0,08; 0,15] | 37  [29; 48] | 0,85 |  |
| **Intervention** | | | - | 211,5 | - | 91,5 | - | 120 | - | 0 | - |
| **Total without intervention** | | | - | 1,092  [869; 1,468] | - | 1,075  [878; 1,367] | - | 1,150  [927; 1,471] | - | 1,025  [833; 1,469] | 0.63 |
| **Total** | | | - | 1303  [1,087; 1,701] | - | 1167  [987; 1,462] | - | 1270  [1,046; 1,572] | - | 1,025  [831; 1,445] | <0.001 |

* Multidomain Intervention; †Polyunsaturated Fatty Acids; ‡Confidence Interval; §p-value of Kruskal-Wallis rank sum test; || Medical Surgery Obstetrics; **GastroIntestinal

Appendix 2.4: Cost (€2018) description according to the cost component and strategies at visit no. 4 (n=1,320)

|  | | | **PFA**^†^ **+ MI*** | | **PFA**^†^ | | **MI*** | | **Control** | |  |
| --- | --- | --- | --- | --- | --- | --- | --- | --- | --- | --- | --- |
|  |  |  | Frequency | Cost | Frequency | Cost | Frequency | Cost | Frequency | Cost | p^§^ |
|  |  |  | Mean  [95% CI^‡^] | Mean  [95% CI^‡^] | Mean  [95% CI^‡^] | Mean  [95% CI^‡^] | Mean  [95% CI^‡^] | Mean  [95% CI^‡^] | Mean  [95% CI^‡^] | Mean  [95% CI^‡^] |  |
| **Inpatient stays** | | | 0.2  [0.15; 0.26] | 750  [541; 1077] | 0.17  [0.12; 0.22] | 753  [519; 1152] | 0.18  [0.12; 0.25] | 701  [472; 1083] | 0.14  [0.1; 0.19] | 547  [357; 907] | 0.41 |
| MSO^\|\|^ <1 day | | 0.06  [0.03; 0.1] | 65  [36; 116] | 0.05  [0.02; 0.08] | 49  [27; 93] | 0.07  [0.04; 0.14] | 68  [38; 120] | 0.04  [0.02; 0.07] | 39  [20; 69] | 0.71 |  |
| MSO^\|\|^ ≥1 day | | 0.13  [0.09; 0.17] | 542  [377; 726] | 0.11  [0.08; 0.16] | 517  [339; 722] | 0.09  [0.06; 0.14] | 408  [266; 647] | 0.1  [0.06; 0.14] | 431  [293; 624] | 0.31 |  |
| Post-acute & rehabilitation care unit | | 0.01  [0; 0.02] | 59  [0; 240] | 0.01  [0; 0.02] | 118  [0; 383] | 0.01  [0; 0.02] | 183  [43; 428] | 0.01  [0; 0.02] | 78  [0; 233] | 0.55 |  |
| Psychiatric unit care | | 0.01  [0; 0.02] | 84  [0; 374] | 0  [0; 0.01] | 68  [0; 205] | 0  [0; 0.01] | 42  [0; 126] | 0  [0; 0] | 0  [0; 0] | 0.57 |  |
| **Emergency** | | | 0.01  [0; 0.02] | 2  [1; 5] | 0.02  [0.01; 0.04] | 3  [1; 8] | 0  [0; 0.01] | 1  [0; 2] | 0.01  [0; 0.02] | 1  [0; 3] | 0.33 |
| **Consultation** | | | 4.23  [3.89; 4.63] | 75  [69; 82] | 4.77  [4.42; 5.2] | 85  [79; 93] | 4.59  [4.15; 5.24] | 81  [74; 93] | 4.9  [4.49; 5.42] | 87  [80; 99] | 0.18 |
| General practitioner | | 2.49  [2.29; 2.72] | 41  [37; 45] | 2.64  [2.42; 2.89] | 43  [39; 47] | 2.46  [2.27; 2.87] | 41  [37; 46] | 2.62  [2.4; 2.82] | 43  [40; 47] | 0.43 |  |
| Specialist | | 1.75  [1.52; 2.02] | 34  [30; 39] | 2.13  [1.86; 2.48] | 42  [37; 48] | 2.13  [1.8; 2.68] | 41  [35; 52] | 2.28  [1.93; 2.73] | 44  [38; 53] | 0.22 |  |
| Cardiologist | 0.32  [0.25; 0.38] | 10  [8; 12] | 0.37  [0.29; 0.45] | 12  [9; 15] | 0.28  [0.21; 0.35] | 9  [7; 11] | 0.3  [0.23; 0.38] | 10  [7; 12] | 0.34 |  |  |
| Psychiatrist & Neurologist | 0.03  [0.01; 0.07] | 1  [0; 2] | 0.08  [0.03; 0.24] | 2  [1; 6] | 0.12  [0.07; 0.21] | 3  [2; 6] | 0.17  [0.08; 0.5] | 4  [2; 14] | 0.023 |  |  |
| Rheumatologist | 0.3  [0.21; 0.42] | 5  [4; 7] | 0.3  [0.21; 0.4] | 5  [3; 6] | 0.29  [0.21; 0.41] | 5  [3; 7] | 0.44  [0.34; 0.57] | 7  [5; 9] | 0.029 |  |  |
| Ophthalmologist | 0.35  [0.26; 0.43] | 6  [4; 7] | 0.26  [0.2; 0.35] | 4  [3; 6] | 0.37  [0.29; 0.48] | 6  [5; 8] | 0.32  [0.25; 0.41] | 5  [4; 7] | 0.39 |  |  |
| Other specialists | 0.75  [0.63; 0.93] | 12  [10; 16] | 1.13  [0.91; 1.43] | 19  [15; 23] | 1.07  [0.81; 1.77] | 18  [13; 29] | 1.06  [0.81; 1.44] | 17  [14; 23] | 0.15 |  |  |
| **Paramedical procedures** | | | 4.28  [2.71; 7.52] | 75  [47; 147] | 2.94  [2.08; 4.09] | 49  [36; 65] | 3.67  [2.56; 6.2] | 53  [38; 76] | 3.87  [2.91; 5.36] | 66  [49; 95] | 0.50 |
| Nurse | | 0.99  [0.38; 2.3] | 24  [4; 120] | 0.24  [0.08; 0.97] | 3  [1; 7] | 0.57  [0.19; 1.77] | 6  [3; 15] | 0.56  [0.27; 1.35] | 11  [5; 36] | 0.90 |  |
| Physiotherapist | | 2.3  [1.65; 3.26] | 48  [33; 70] | 2.47  [1.85; 3.43] | 46  [34; 62] | 2.53  [1.85; 3.61] | 46  [33; 63] | 2.76  [2.1; 3.85] | 53  [39; 77] | 0.72 |  |
| Other | | 0.99  [0.39; 2.34] | 3  [1; 6] | 0.24  [0.07; 0.85] | 1  [0; 2] | 0.57  [0.2; 1.73] | 2  [1; 5] | 0.56  [0.26; 1.28] | 1  [1; 4] | 0.91 |  |
| **Medical procedures** | | | 4.25  [3.66; 5.47] | 33  [29; 39] | 4.63  [3.98; 5.33] | 49  [42; 61] | 3.82  [3.22; 4.66] | 29  [25; 35] | 3.92  [3.42; 4.49] | 40  [34; 47] | 0.001 |
| Laboratory tests | | 3.7  [3.06; 4.65] | 15  [13; 19] | 3.92  [3.38; 4.56] | 27  [21; 37] | 3.38  [2.82; 4.18] | 14  [12; 18] | 3.3  [2.77; 3.81] | 19  [16; 23] | 0.016 |  |
| Imaging | | 0.43  [0.34; 0.52] | 14  [11; 17] | 0.55  [0.45; 0.65] | 19  [15; 24] | 0.37  [0.29; 0.46] | 13  [10; 17] | 0.48  [0.39; 0.58] | 17  [14; 21] | 0.046 |  |
| Other | | 0.13  [0.09; 0.19] | 4  [3; 6] | 0.16  [0.11; 0.23] | 4  [2; 5] | 0.08  [0.05; 0.11] | 2  [1; 4] | 0.13  [0.09; 0.18] | 4  [3; 6] | 0.31 |  |
| **Transportation** | | | 0.42  [0.03; 2.34] | 10  [1; 48] | 0.06  [0.01; 0.14] | 3  [1; 9] | 0.16  [0.03; 0.66] | 1  [1; 3] | 0.09  [0.01; 0.47] | 1  [0; 4] | 0.59 |
| **Medications** | | | 5.72  [5.28; 6.21] | 367  [322; 430] | 5.31  [4.92; 5.76] | 327  [290; 377] | 5.19  [4.83; 5.6] | 386  [333; 470] | 5.53  [5.18; 5.95] | 368  [325; 444] | 0.45 |
| Cardiovascular system | | 1.81  [1.64; 2.05] | 101  [89; 115] | 1.61  [1.46; 1.8] | 89  [79; 100] | 1.46  [1.31; 1.62] | 89  [76; 107] | 1.47  [1.32; 1.63] | 82  [71; 93] | 0.23 |  |
| GI** tract and metabolism | | 1.13  [0.99; 1.31] | 60  [50; 72] | 1.04  [0.9; 1.18] | 57  [48; 67] | 0.94  [0.83; 1.07] | 51  [43; 61] | 1.06  [0.93; 1.2] | 58  [48; 70] | 0.79 |  |
| Nervous system | | 1.88  [1.69; 2.08] | 152  [121; 210] | 1.8  [1.57; 2.06] | 126  [100; 168] | 1.68  [1.5; 1.87] | 174  [128; 266] | 2.01  [1.83; 2.19] | 165  [133; 228] | 0.013 |  |
| Anti-depressants | 0.89  [0.77; 1.07] | 54  [46; 66] | 0.87  [0.74; 1] | 55  [46; 67] | 1.11  [0.96; 1.25] | 72  [61; 89] | 0.98  [0.85; 1.15] | 63  [51; 75] | 0.10 |  |  |
| Anxiolytics | 0.21  [0.15; 0.26] | 12  [9; 18] | 0.16  [0.11; 0.2] | 8  [6; 12] | 0.28  [0.22; 0.34] | 15  [11; 20] | 0.19  [0.14; 0.25] | 11  [8; 16] | 0.031 |  |  |
| Hypnotics and sedatives | 0.17  [0.12; 0.22] | 4  [3; 8] | 0.13  [0.1; 0.18] | 3  [2; 4] | 0.18  [0.14; 0.23] | 4  [3; 5] | 0.14  [0.1; 0.18] | 3  [2; 5] | 0.42 |  |  |
| Other nervous system  drugs | 0.44  [0.36; 0.53] | 3  [2; 4] | 0.49  [0.41; 0.6] | 2  [1; 3] | 0.54  [0.44; 0.64] | 3  [2; 4] | 0.53  [0.44; 0.62] | 4  [3; 5] | 0.12 |  |  |
| Other drugs | | 0.08  [0.05; 0.11] | 36  [30; 44] | 0.08  [0.05; 0.11] | 41  [33; 52] | 0.11  [0.08; 0.15] | 51  [40; 66] | 0.13  [0.09; 0.17] | 45  [35; 57] | 0.87 |  |
| **Intervention** | | | - | 211.5 | - | 91.5 | - | 120 | - | 0 | - |
| **Total without intervention** | | | - | 1312  [1,079; 1,705] | - | 1,270  [1,029; 1,644] | - | 1,253  [990; 1637] | - | 1110  [919; 1,503] | 0.63 |
| **Total** | | | - | 1524  [1,281; 1,821] | - | 1361  [1,102; 1,725] | - | 1373  [1,121; 1,764] | - | 1110  [914; 1,488] | <0.001 |

* Multidomain Intervention; †Polyunsaturated Fatty Acids; ‡Confidence Interval; §p-value of Kruskal-Wallis rank sum test; || Medical Surgery Obstetrics; **GastroIntestinal

|  | | | **PFA**^†^ **+ MI*** | | **PFA**^†^ | | **MI*** | | **Control** | |  |
| --- | --- | --- | --- | --- | --- | --- | --- | --- | --- | --- | --- |
|  |  |  | Frequency | Cost | Frequency | Cost | Frequency | Cost | Frequency | Cost | p^§^ |
|  |  |  | Mean  [95% CI^‡^] | Mean  [95% CI^‡^] | Mean  [95% CI^‡^] | Mean  [95% CI^‡^] | Mean  [95% CI^‡^] | Mean  [95% CI^‡^] | Mean  [95% CI^‡^] | Mean  [95% CI^‡^] |  |
| **Inpatient stays** | | | 0.18  [0.14; 0.26] | 988  [628; 1640] | 0.16  [0.11; 0.22] | 855  [502; 2274] | 0.19  [0.13; 0.27] | 897  [554; 1687] | 0.14  [0.1; 0.21] | 610  [336; 1149] | 0.38 |
| MSO^\|\|^ <1 day | | 0.04  [0.02; 0.07] | 42  [24; 77] | 0.04  [0.02; 0.06] | 44  [22; 77] | 0.08  [0.04; 0.15] | 82  [45; 140] | 0.08  [0.05; 0.12] | 79  [47; 127] | 0.49 |  |
| MSO^\|\|^≥1 day | | 0.12  [0.08; 0.17] | 520  [377; 792] | 0.12  [0.08; 0.17] | 513  [361; 742] | 0.09  [0.06; 0.13] | 422  [280; 636] | 0.06  [0.03; 0.09] | 284  [173; 472] | 0.10 |  |
| Post-acute & rehabilitation care unit | | 0.01  [0; 0.03] | 184  [50; 468] | 0.01  [0; 0.02] | 298  [0; 1430] | 0.02  [0.01; 0.06] | 393  [113; 1038] | 0.01  [0; 0.02] | 246  [64; 653] | 0.70 |  |
| Psychiatric unit care | | 0.01  [0; 0.02] | 241  [42; 712] | 0  [0; 0] | 0  [0; 0] | 0  [0; 0] | 0  [0; 0] | 0  [0; 0] | 0  [0; 0] | 0.029 |  |
| **Emergency** | | | 0.01  [0; 0.03] | 2  [0; 6] | 0.01  [0; 0.02] | 2  [0; 4] | 0  [0; 0.01] | 1  [0; 2] | 0.01  [0; 0.02] | 2  [1; 5] | 0.63 |
| **Consultation** | | | 4.34  [3.93; 4.87] | 77  [70; 87] | 4.73  [4.32; 5.37] | 83  [76; 94] | 4.17  [3.8; 4.62] | 73  [67; 81] | 4.59  [4.22; 5.1] | 82  [74; 91] | 0.047 |
| General practitioner | | 2.51  [2.31; 2.78] | 41  [38; 45] | 2.67  [2.47; 2.91] | 44  [41; 48] | 2.44  [2.21; 2.66] | 40  [37; 44] | 2.45  [2.26; 2.67] | 40  [37; 44] | 0.16 |  |
| Specialist | | 1.82  [1.53; 2.34] | 36  [30; 44] | 2.06  [1.75; 2.68] | 39  [34; 49] | 1.73  [1.49; 2.08] | 33  [28; 39] | 2.14  [1.83; 2.57] | 41  [35; 50] | 0.16 |  |
| Cardiologist | 0.28  [0.21; 0.36] | 9  [7; 12] | 0.3  [0.23; 0.38] | 10  [8; 12] | 0.24  [0.18; 0.33] | 8  [6; 11] | 0.26  [0.19; 0.35] | 8  [6; 11] | 0.17 |  |  |
| Psychiatrist & Neurologist | 0.1  [0.05; 0.23] | 3  [1; 6] | 0.05  [0.02; 0.11] | 1  [1; 3] | 0.08  [0.04; 0.15] | 2  [1; 4] | 0.2  [0.1; 0.49] | 5  [3; 12] | 0.06 |  |  |
| Rheumatologist | 0.28  [0.2; 0.41] | 5  [3; 6] | 0.29  [0.22; 0.4] | 5  [4; 6] | 0.26  [0.18; 0.37] | 4  [3; 6] | 0.32  [0.25; 0.42] | 5  [4; 7] | 0.34 |  |  |
| Ophthalmologist | 0.27  [0.2; 0.35] | 4  [3; 6] | 0.32  [0.25; 0.42] | 5  [4; 7] | 0.41  [0.31; 0.56] | 7  [5; 9] | 0.37  [0.27; 0.47] | 6  [5; 8] | 0.57 |  |  |
| Other specialists | 0.89  [0.67; 1.37] | 15  [10; 22] | 1.1  [0.85; 1.66] | 18  [14; 28] | 0.75  [0.58; 1.15] | 12  [10; 18] | 0.99  [0.76; 1.34] | 16  [13; 23] | 0.027 |  |  |
| **Paramedical procedures** | | | 3.1  [2.04; 4.72] | 59  [41; 97] | 8.76  [3.15; 22.43] | 102  [58; 276] | 3.06  [2.11; 4.76] | 52  [36; 75] | 3.11  [2.26; 4.66] | 56  [40; 80] | 0.36 |
| Nurse | | 0.27  [0.08; 0.87] | 9  [1; 39] | 3.23  [0.46; 9.97] | 45  [10; 177] | 0.3  [0.11; 0.97] | 5  [2; 11] | 0.32  [0.11; 0.94] | 6  [2; 22] | 0.27 |  |
| Physiotherapist | | 2.55  [1.8; 3.77] | 49  [35; 67] | 2.3  [1.72; 2.99] | 48  [35; 64] | 2.46  [1.66; 3.49] | 47  [32; 66] | 2.47  [1.82; 3.36] | 50  [36; 67] | 0.61 |  |
| Other | | 0.27  [0.08; 1.11] | 1  [0; 3] | 3.23  [0.47; 12.83] | 9  [1; 25] | 0.3  [0.11; 0.87] | 1  [0; 2] | 0.32  [0.11; 0.99] | 1  [0; 2] | 0.17 |  |
| **Medical procedures** | | | 3.77  [3.26; 4.34] | 32  [28; 38] | 3.84  [3.34; 4.43] | 40  [35; 47] | 3.82  [3.26; 4.5] | 32  [26; 40] | 3.48  [3.03; 4.05] | 40  [34; 47] | 0.07 |
| Laboratory tests | | 3.27  [2.78; 3.91] | 16  [13; 20] | 3.25  [2.81; 3.81] | 19  [16; 22] | 3.42  [2.91; 4.1] | 17  [14; 30] | 2.89  [2.43; 3.34] | 20  [16; 25] | 0.45 |  |
| Imaging | | 0.38  [0.3; 0.46] | 13  [10; 16] | 0.46  [0.37; 0.57] | 17  [14; 21] | 0.35  [0.27; 0.45] | 12  [9; 16] | 0.47  [0.37; 0.58] | 16  [13; 21] | 0.16 |  |
| Other | | 0.12  [0.09; 0.18] | 4  [3; 6] | 0.13  [0.09; 0.19] | 4  [2; 6] | 0.05  [0.03; 0.08] | 2  [1; 3] | 0.13  [0.09; 0.18] | 4  [2; 6] | 0.011 |  |
| **Transportation** | | | 0.25  [0.06; 0.67] | 7  [2; 20] | 0.04  [0.01; 0.11] | 3  [0; 10] | 0.11  [0.05; 0.3] | 3  [1; 7] | 0.05  [0.02; 0.15] | 2  [1; 7] | 0.66 |
| **Medications** | | | 6  [5.57; 6.47] | 424  [371; 520] | 5.39  [5.01; 5.84] | 342  [307; 385] | 5.1  [4.73; 5.49] | 414  [347; 561] | 5.5  [5.05; 5.89] | 365  [325; 432] | 0.29 |
| Cardiovascular system | | 1.91  [1.7; 2.13] | 108  [94; 124] | 1.58  [1.43; 1.77] | 92  [81; 104] | 1.46  [1.31; 1.63] | 83  [72; 97] | 1.4  [1.26; 1.55] | 75  [66; 87] | 0.005 |  |
| GI** tract and metabolism | | 1.15  [1.01; 1.31] | 62  [51; 76] | 1.06  [0.95; 1.2] | 62  [51; 73] | 0.94  [0.82; 1.07] | 49  [42; 59] | 1.04  [0.92; 1.18] | 57  [46; 67] | 0.46 |  |
| Nervous system | | 1.95  [1.75; 2.16] | 187  [142; 272] | 1.85  [1.67; 2.09] | 129  [107; 166] | 1.64  [1.45; 1.83] | 212  [151; 350] | 2.05  [1.84; 2.28] | 175  [139; 230] | 0.019 |  |
| Anti-depressants | 1  [0.88; 1.17] | 67  [54; 87] | 0.9  [0.76; 1.03] | 59  [49; 71] | 1.06  [0.92; 1.21] | 70  [58; 86] | 1.01  [0.88; 1.16] | 58  [49; 71] | 0.56 |  |  |
| Anxiolytics | 0.22  [0.17; 0.29] | 12  [9; 16] | 0.18  [0.13; 0.23] | 10  [7; 14] | 0.25  [0.19; 0.31] | 13  [10; 17] | 0.2  [0.14; 0.25] | 10  [7; 14] | 0.26 |  |  |
| Hypnotics and sedatives | 0.16  [0.12; 0.22] | 4  [3; 6] | 0.14  [0.1; 0.18] | 3  [3; 5] | 0.16  [0.12; 0.21] | 4  [3; 5] | 0.15  [0.11; 0.19] | 4  [3; 5] | 0.91 |  |  |
| Other nervous system  drugs | 0.52  [0.43; 0.62] | 3  [2; 4] | 0.5  [0.41; 0.59] | 2  [2; 3] | 0.55  [0.45; 0.66] | 3  [2; 4] | 0.56  [0.47; 0.67] | 3  [2; 4] | 0.77 |  |  |
| Other drugs | | 0.1  [0.06; 0.12] | 48  [38; 65] | 0.08  [0.05; 0.11] | 43  [35; 53] | 0.1  [0.06; 0.14] | 50  [40; 65] | 0.11  [0.08; 0.15] | 41  [33; 54] | 0.95 |  |
| **Intervention** | | | - | 211.5 | - | 91.5 | - | 120 | - | 0 | - |
| **Total without intervention** | | | - | 1,589  [1,209; 2,345] | - | 1,426  [1,063; 2,612] | - | 1,472  [1,082; 2,173] | - | 1,157  [869; 1,704] | 0.38 |
| **Total** | | | - | 1,800  [1,391; 2,529] | - | 1,517  [1,158; 2,703] | - | 1592  [1,217; 2,376] | - | 1,157  [891; 1,681] | <0.001 |

Appendix 2.5: Cost (€2018) description according to the cost component and strategies at visit no. 5 (n=1,320)

* Multidomain Intervention; †Polyunsaturated Fatty Acids; ‡Confidence Interval; §p-value of Kruskal-Wallis rank sum test; || Medical Surgery Obstetrics; **GastroIntestinal

|  | | | **PFA**^†^ **+ MI*** | | **PFA**^†^ | | **MI*** | | **Control** | |  |  |
| --- | --- | --- | --- | --- | --- | --- | --- | --- | --- | --- | --- | --- |
|  |  |  | Frequency | Cost | Frequency | Cost | Frequency | Cost | Frequency | Cost |  | p^§^ |
|  |  |  | Mean  [95% CI^‡^] | Mean  [95% CI^‡^] | Mean  [95% CI^‡^] | Mean  [95% CI^‡^] | Mean  [95% CI^‡^] | Mean  [95% CI^‡^] | Mean  [95% CI^‡^] | Mean  [95% CI^‡^] |  |  |
| **Inpatient stays** | | | 0.21  [0.15; 0.27] | 1064  [688; 1676] | 0.16  [0.11; 0.21] | 881  [541; 2104] | 0.16  [0.12; 0.22] | 1030  [612; 1873] | 0.21  [0.15; 0.29] | 1025  [738; 1457] |  | 0.23 |
| MSO^\|\|^ <1 day | | 0.05  [0.03; 0.09] | 48  [26; 84] | 0.04  [0.02; 0.06] | 34  [17; 64] | 0.05  [0.03; 0.08] | 51  [28; 89] | 0.05  [0.02; 0.12] | 43  [20; 93] |  | 0.82 |  |
| MSO^\|\|^ ≥1 day | | 0.15  [0.11; 0.2] | 663  [478; 973] | 0.11  [0.07; 0.15] | 475  [329; 669] | 0.09  [0.06; 0.13] | 374  [249; 550] | 0.14  [0.1; 0.19] | 694  [481; 966] |  | 0.14 |  |
| Post-acute & rehabilitation care unit | | 0.01  [0; 0.02] | 230  [24; 731] | 0.01  [0; 0.02] | 139  [32; 392] | 0.02  [0; 0.03] | 240  [75; 548] | 0.02  [0.01; 0.03] | 205  [78; 455] |  | 0.68 |  |
| Psychiatric unit care | | 0  [0; 0.01] | 124  [0; 371] | 0  [0; 0.01] | 233  [0; 700] | 0.01  [0; 0.03] | 364  [0; 1051] | 0  [0; 0.01] | 83  [0; 250] |  | 0.89 |  |
| **Emergency** | | | 0.01  [0; 0.02] | 2  [0; 4] | 0  [0; 0.01] | 1  [0; 2] | 0.01  [0; 0.03] | 2  [0; 6] | 0.01  [0; 0.02] | 2  [0; 3] |  | 0.75 |
| **Consultation** | | | 4.29  [3.95; 4.78] | 76  [70; 84] | 5.21  [4.81; 5.74] | 92  [85; 102] | 4.88  [4.43; 5.86] | 87  [78; 103] | 4.98  [4.5; 5.56] | 88  [80; 99] |  | 0.06 |
| General practitioner | | 2.48  [2.29; 2.71] | 41  [38; 45] | 2.82  [2.6; 3.11] | 47  [43; 51] | 2.66  [2.42; 3.08] | 44  [40; 52] | 2.57  [2.36; 2.81] | 42  [39; 46] |  | 0.11 |  |
| Specialist | | 1.81  [1.57; 2.16] | 35  [31; 43] | 2.39  [2.05; 2.85] | 46  [40; 53] | 2.22  [1.85; 3.22] | 43  [36; 57] | 2.4  [2.04; 2.98] | 45  [38; 56] |  | 0.15 |  |
| Cardiologist | 0.28  [0.22; 0.34] | 9  [7; 11] | 0.35  [0.28; 0.44] | 11  [9; 14] | 0.32  [0.25; 0.44] | 10  [8; 14] | 0.23  [0.17; 0.3] | 7  [6; 10] |  | 0.09 |  |  |
| Psychiatrist & Neurologist | 0.1  [0.05; 0.17] | 3  [2; 4] | 0.09  [0.05; 0.21] | 2  [1; 6] | 0.11  [0.06; 0.2] | 3  [1; 5] | 0.21  [0.07; 0.56] | 5  [2; 15] |  | 0.96 |  |  |
| Rheumatologist | 0.34  [0.25; 0.54] | 6  [4; 9] | 0.32  [0.25; 0.46] | 5  [4; 8] | 0.34  [0.24; 0.47] | 6  [4; 8] | 0.44  [0.31; 0.82] | 7  [5; 14] |  | 0.68 |  |  |
| Ophthalmologist | 0.35  [0.27; 0.43] | 6  [5; 7] | 0.32  [0.25; 0.42] | 5  [4; 7] | 0.34  [0.25; 0.45] | 6  [4; 8] | 0.39  [0.3; 0.52] | 6  [5; 9] |  | 0.15 |  |  |
| Other specialists | 0.74  [0.6; 0.94] | 12  [10; 16] | 1.3  [1.06; 1.74] | 21  [17; 29] | 1.12  [0.81; 2.17] | 19  [13; 33] | 1.13  [0.9; 1.48] | 19  [15; 24] |  | 0.001 |  |  |
| **Paramedical procedures** | | | 2.64  [1.95; 3.63] | 43  [29; 71] | 6.11  [3.33; 16.97] | 74  [51; 113] | 3.21  [2.35; 4.6] | 62  [44; 95] | 4.42  [3.34; 6.69] | 90  [60; 201] |  | 0.06 |
| Nurse | | 0.27  [0.15; 0.45] | 4  [2; 8] | 1.62  [0.24; 6.55] | 8  [3; 27] | 0.31  [0.14; 0.63] | 12  [2; 50] | 0.56  [0.24; 1.44] | 27  [6; 123] |  | 0.73 |  |
| Physiotherapist | | 2.11  [1.44; 3.3] | 38  [25; 65] | 2.88  [2.09; 3.81] | 62  [44; 89] | 2.59  [1.85; 3.67] | 50  [33; 69] | 3.31  [2.45; 4.4] | 61  [46; 81] |  | 0.024 |  |
| Other | | 0.27  [0.15; 0.47] | 1  [0; 1] | 1.62  [0.28; 8.7] | 4  [1; 18] | 0.31  [0.15; 0.66] | 1  [0; 2] | 0.56  [0.23; 1.62] | 1  [1; 4] |  | 0.78 |  |
| **Medical procedures** | | | 3.88  [3.34; 4.59] | 32  [26; 42] | 4.05  [3.43; 5.39] | 45  [38; 53] | 4.22  [3.6; 5.07] | 37  [32; 45] | 4.09  [3.46; 4.79] | 41  [34; 51] |  | 0.06 |
| Laboratory tests | | 3.42  [2.9; 4.11] | 16  [13; 22] | 3.42  [2.85; 4.73] | 22  [19; 27] | 3.69  [3.08; 4.49] | 18  [15; 24] | 3.38  [2.8; 4.11] | 20  [16; 27] |  | 0.047 |  |
| Imaging | | 0.37  [0.28; 0.48] | 13  [10; 17] | 0.48  [0.39; 0.6] | 18  [15; 24] | 0.44  [0.35; 0.56] | 15  [12; 19] | 0.54  [0.43; 0.68] | 17  [13; 22] |  | 0.28 |  |
| Other | | 0.09  [0.06; 0.14] | 3  [2; 6] | 0.14  [0.1; 0.2] | 5  [3; 7] | 0.1  [0.06; 0.14] | 3  [2; 5] | 0.17  [0.11; 0.24] | 4  [3; 6] |  | 0.18 |  |
| **Transportation** | | | 0.14  [0.02; 0.72] | 11  [1; 50] | 0.06  [0.02; 0.16] | 1  [0; 3] | 0.32  [0.09; 1.13] | 32  [2; 149] | 0.17  [0.04; 0.51] | 5  [1; 21] |  | 0.20 |
| **Medications** | | | 5.89  [5.45; 6.38] | 436  [389; 504] | 5.68  [5.28; 6.06] | 399  [356; 465] | 5.24  [4.85; 5.6] | 455  [383; 562] | 5.55  [5.14; 5.99] | 383  [341; 453] |  | 0.45 |
| Cardiovascular system | | 1.8  [1.6; 2.02] | 111  [96; 127] | 1.61  [1.47; 1.78] | 99  [86; 112] | 1.48  [1.33; 1.66] | 103  [88; 142] | 1.39  [1.24; 1.55] | 80  [69; 93] |  | 0.015 |  |
| GI** tract and metabolism | | 1.15  [1; 1.33] | 73  [60; 95] | 1.14  [1.01; 1.28] | 69  [58; 82] | 0.97  [0.85; 1.1] | 60  [49; 74] | 1.09  [0.95; 1.23] | 59  [50; 71] |  | 0.45 |  |
| Nervous system | | 1.97  [1.76; 2.19] | 187  [148; 247] | 1.91  [1.72; 2.14] | 155  [123; 208] | 1.69  [1.52; 1.89] | 210  [156; 314] | 2.08  [1.89; 2.3] | 181  [148; 240] |  | 0.14 |  |
| Anti-depressants | 0.98  [0.86; 1.12] | 65  [55; 80] | 1.02  [0.87; 1.14] | 76  [63; 98] | 1.09  [0.95; 1.25] | 82  [67; 100] | 1  [0.86; 1.14] | 63  [53; 77] |  | 0.31 |  |  |
| Anxiolytics | 0.2  [0.16; 0.26] | 11  [8; 14] | 0.2  [0.15; 0.26] | 11  [8; 15] | 0.26  [0.21; 0.32] | 16  [12; 20] | 0.19  [0.14; 0.25] | 11  [8; 15] |  | 0.08 |  |  |
| Hypnotics and sedatives | 0.17  [0.13; 0.22] | 4  [3; 5] | 0.14  [0.11; 0.19] | 4  [3; 5] | 0.18  [0.13; 0.23] | 4  [3; 6] | 0.14  [0.11; 0.18] | 4  [3; 5] |  | 0.72 |  |  |
| Other nervous system  drugs | 0.51  [0.43; 0.6] | 3  [2; 4] | 0.57  [0.48; 0.66] | 3  [2; 4] | 0.55  [0.46; 0.66] | 3  [2; 5] | 0.55  [0.47; 0.66] | 4  [3; 5] |  | 0.81 |  |  |
| Other drugs | | 0.09  [0.06; 0.12] | 47  [39; 62] | 0.09  [0.06; 0.13] | 59  [46; 81] | 0.1  [0.07; 0.14] | 58  [46; 76] | 0.12  [0.08; 0.16] | 45  [36; 57] |  | 0.71 |  |
| **Intervention** | | | - | 211.5 | - | 91.5 | - | 120 | - | 0 |  | - |
| **Total without intervention** | | | - | 1,664  [1,284; 2,345] | - | 1,494  [1,133; 2,532] | - | 1,704  [1,252; 2,557] | - | 1,634  [1,303; 2,081] |  | 0.86 |
| **Total** | | | - | 1,876  [1,480; 2,518] | - | 1,585  [1,218; 2,421] | - | 1,824  [1,403; 2,779] | - | 1634  [1,328; 2,139] |  | <0.001 |

Appendix 2.6: Cost (€2018) description according to the cost component and strategies at visit no. 6 (n=1,320)

*Multidomain Intervention; †Polyunsaturated Fatty Acids; ‡Confidence Interval; §p-value of Kruskal-Wallis rank sum test; || Medical Surgery Obstetrics; **GastroIntestinal
