# Supplementary material for: The Cost-Effectiveness of Three Prevention Strategies in Alzheimer's Disease: Results from the Multidomain Alzheimer Preventive Trial (MAPT)
Source: J Prev Alzheimers Dis. 2021 Aug 2;8(4):425–35. doi: 10.14283/jpad.2021.47 (PMC12280784; doi:10.14283/jpad.2021.47)
Supplement: Supplementary file 3 — Appendix 3- Table A3. Three-year costs, medical outcomes, effectiveness and cost-effectiveness (N=1,525) [file mmc3.docx]

**Appendix 3- Table A3. Three-year costs, medical outcomes, effectiveness and cost-effectiveness (N=1,525)**

| **N=**  **1,525** | **Costs (€2018)*** | **Z Score at 3 years*** | **Z Score Δ between 3 years and baseline** | **Frequency of aggravations** | **Percent of aggravations** | **Δ between intervention placebo groups** | | | | **ICER^\|\|^** | | |
| --- | --- | --- | --- | --- | --- | --- | --- | --- | --- | --- | --- | --- |
|  | Mean  [95% CI^†^] | Mean  [95% CI^†^] | Mean  [95% CI^†^] | N  [95% CI^†^] | %  [95% CI^†^] | Cost Δ | Z Score Δ | Δ N of no aggravation | Δ % of no aggravation | Z Score Δ | Δ N of no aggravation | Δ % of no aggravation |
| Placebo | 7,084  [6,848; 7,331] | -0.051  [-0.138; 0.036] | -0.069  [-0.104; -0.041] | 323  [309; 336] | 85  [81.3; 88.4] | - | - | - | - | - | - | - |
| PFA^§^ | 8,321  [8,045; 8,592] | -0.029  [-0.112; 0.059] | -0.057  [-0.094; -0.035] | 325  [312; 339] | 85.3  [81.2; 88.5] | 1,237 | 0.011 | 2 | 0.30 | 11,1720 | 618 | 4,098 |
| MI^‡^ | 8,789  [8,496; 9,091] | 0.008  [-0.088; 0.092] | 0.011  [-0.023; 0.041] | 339  [326; 352] | 86.9  [83.6; 90.3] | 1,705 | 0.079 | 13 | 1.92 | 21,543 | 107 | 887 |
| PFA^§^ + MI^‡^ | 9,070  [8,761; 9,369] | -0.019  [-0.107; 0.064] | 0.024  [-0.005; 0.049] | 335  [323; 347] | 89.6  [86.4; 92.8] | 1,986 | 0.093 | 12 | 4.57 | 21,443 | 166 | 434 |

*Using fitted value from the multivariate analysis; †Confidence Interval; ‡Multidomain Intervention; §Polyunsaturated Fatty Acids; ||Incremental Cost-Effectiveness Ratio
