# Supplementary material for: The Cost-Effectiveness of Three Prevention Strategies in Alzheimer's Disease: Results from the Multidomain Alzheimer Preventive Trial (MAPT)
Source: J Prev Alzheimers Dis. 2021 Aug 2;8(4):425–35. doi: 10.14283/jpad.2021.47 (PMC12280784; doi:10.14283/jpad.2021.47)
Supplement: Supplementary file 4 — Appendix 4- Figure A4: Acceptability curves of intervention strategies vs. placebo using different effectiveness criteria [file mmc4.docx]

**Appendix 4- Figure A4: Acceptability curves of intervention strategies vs. placebo using different effectiveness criteria**

**Appendix 4.b: Acceptability curves of intervention strategies vs. placebo using percentage of participants with no aggravation between 3 years and baseline**

**Appendix 4.a: Acceptability curves of intervention strategies vs. placebo using changes in Z score between 3 years and baseline**

**
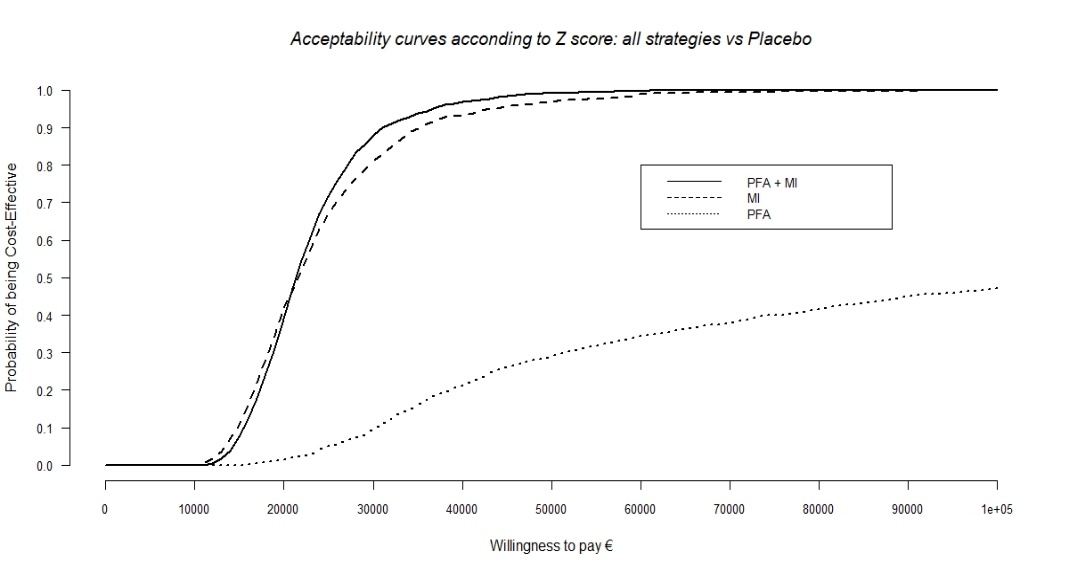

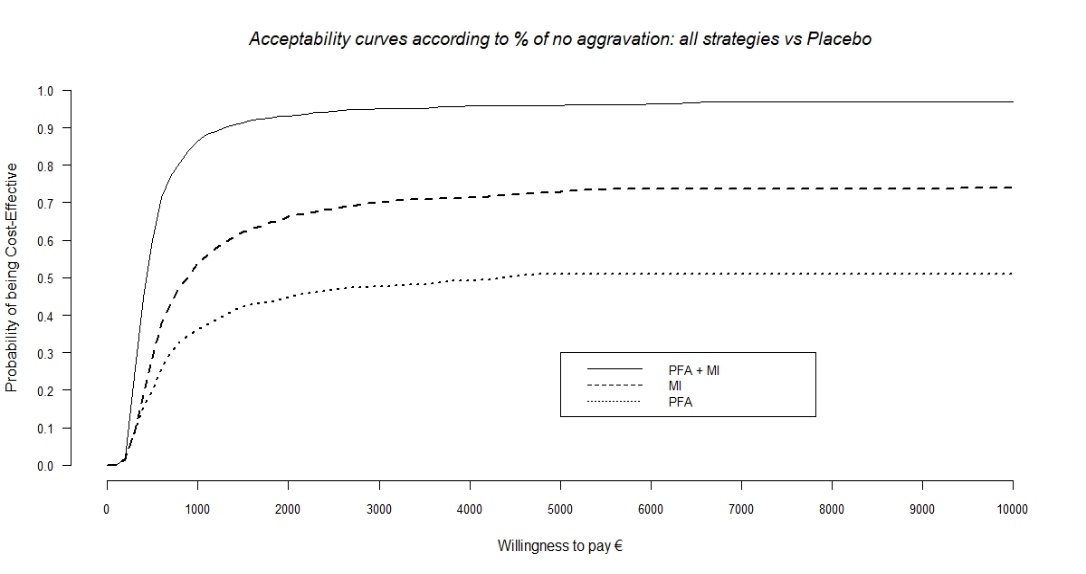
**

**
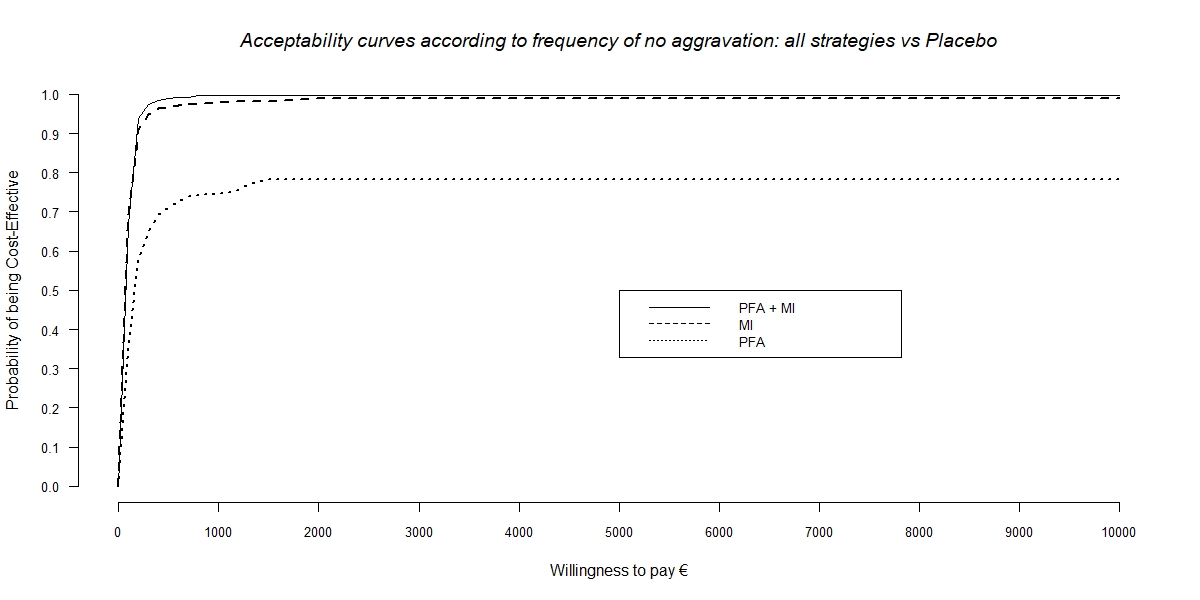
**

**Appendix 4.c: Acceptability curves of intervention strategies vs. placebo using number of participant with no aggravation between 3 years and baseline**
