# Supplementary material for: The Cost-Effectiveness of Three Prevention Strategies in Alzheimer's Disease: Results from the Multidomain Alzheimer Preventive Trial (MAPT)
Source: J Prev Alzheimers Dis. 2021 Aug 2;8(4):425–35. doi: 10.14283/jpad.2021.47 (PMC12280784; doi:10.14283/jpad.2021.47)

**Appendix 5 – Table A5. Three years costs, medical outcomes, effectiveness and cost-effectiveness (N=1,320)**

| **N=**  **1320** | **Costs (€)*** | **Z Score at 3 years*** | **ΔZ Score between 3 years and baseline** | **Percent of no aggravation** | **Δ between intervention placebo groups** | | | **ICER** | |
| --- | --- | --- | --- | --- | --- | --- | --- | --- | --- |
|  | Mean  [95% CI] | Mean  [95% CI] | Mean  [95% CI] | %  [95% CI] | ΔCosts | ΔZ Score | Δ % of no aggravation | ΔZ Score | Δ % of no aggravation |
| Placebo | 7206  [6944 ; 7473] | 0.020  [-0.064 ; 0.110] | -0.052  [-0.090 ; -0.021] | 86.2  [81.1 ; 89.2] | - | - | - | - | - |
| PFA | 8474  [8179 ; 8751] | -0.008  [-0.103 ; 0.080] | -0.047  [-0.084 ; -0.021] | 85.6  [80.9 ; 88.9] | 1268 | 0.005 | -0.6 | 253600 | -2113 |
| MI | 8833  [8528 ; 9152] | 0.074  [-0.018 ; 0.164] | 0.027  [-0.009 ; 0.063] | 87.9  [83.9 ; 90.9] | 1627 | 0.079 | 1.7 | 20595 | 957 |
| PFA + MI | 9052  [8731 ; 9370] | 0.048  [-0.044 ; 0.139] | 0.042  [0.015 ; 0.067] | 91.0  [86.6 ; 93.3] | 1846 | 0.094 | 4.8 | 19638 | 385 |

Costs expressed in €2018; *Using fitted value coming from the multivariate analysis CI: Confident Intervals; MI: Multidomain Intervention; ICER: Incremental Cost-Effectiveness ratio

Appendix 5 – Figure A-5 Confidence ellipses of intervention strategies versus placebo (N=1320)


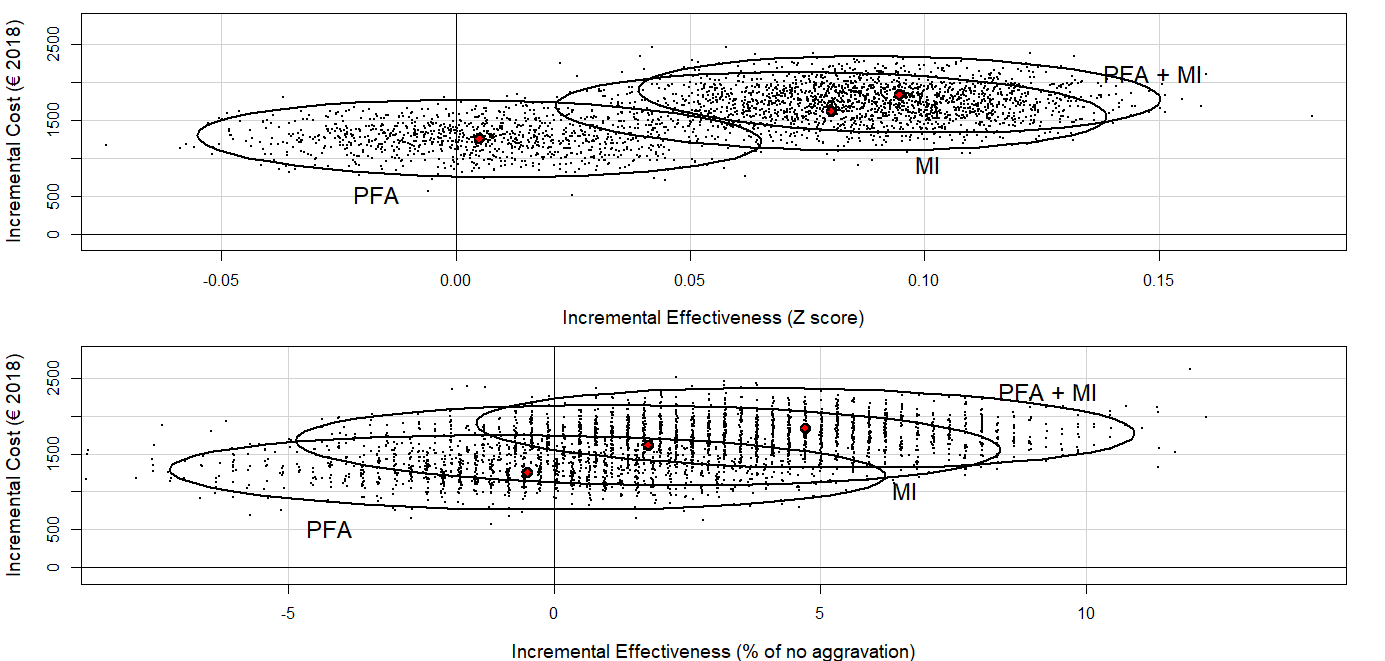

Supplement: Supplementary file 5 — Appendix 5 - Table A5. Three years costs, medical outcomes, effectiveness and cost-effectiveness (N=1,320) [file mmc5.docx]
